# Disposition of Oral Nalbuphine and Its Metabolites in Healthy Subjects and Subjects with Hepatic Impairment: Preliminary Modeling Results Using a Continuous Intestinal Absorption Model with Enterohepatic Recirculation

**DOI:** 10.3390/metabo14090471

**Published:** 2024-08-27

**Authors:** Swati Nagar, Amale Hawi, Thomas Sciascia, Ken Korzekwa

**Affiliations:** 1 Department of Pharmaceutical Sciences, Temple University School of Pharmacy, Philadelphia, PA 19130, USA; swati.nagar@temple.edu; 2 A. Hawi Consulting, Ridgefield, CT 06877, USA; ahawi@ahawi.com; 3 Trevi Therapeutics, New Haven, CT 06510, USA; thomas.sciascia@trevitherapeutics.com

**Keywords:** nalbuphine, hepatic impairment, pharmacokinetics, mechanistic modeling, enterohepatic recycling, drug metabolism

## Abstract

Nalbuphine (NAL) is a mixed κ-agonist/μ-antagonist opioid with extensive first-pass metabolism. A phase 1 open-label study was conducted to characterize the pharmacokinetics (PKs) of NAL and select metabolites following single oral doses of NAL extended-release tablets in subjects with mild, moderate, and severe hepatic impairment (Child–Pugh A, B, and C, respectively) compared to healthy matched subjects. NAL exposures were similar for subjects with mild hepatic impairment as compared to healthy subjects and nearly three-fold and eight-fold higher in subjects with moderate and severe hepatic impairment, respectively. Datasets obtained for healthy, moderate, and severe hepatic impaired groups were modeled with a mechanistic model that incorporated NAL hepatic metabolism and enterohepatic recycling of NAL and its glucuronidated metabolites. The mechanistic model includes a continuous intestinal absorption model linked to semi-physiological liver–gallbladder–compartmental PK models based on partial differential equations (termed the PDE-EHR model). In vitro studies indicated that cytochromes P450 CYP2C9 and CYP2C19 are the major CYPs involved in NAL oxidation, with glucuronidation mainly catalyzed by UGT1A8 and UGT2B7 isozymes. Complex formation and elimination kinetics of NAL and four main metabolites was well predicted by PDE-EHR. The model is expected to improve predictions of drug interactions and complex drug disposition.

## 1. Introduction

Nalbuphine (NAL) is a mixed κ-agonist/μ-antagonist opioid currently being developed as an oral extended-release (ER) tablet for the treatment of chronic cough conditions [1]. The parenteral formulation, Nalbuphine HCl injection, is an approved drug indicated for the management of severe pain [2]. NAL is a low molecular weight, water-soluble molecule with low protein binding (50%). Following oral administration, NAL ER plasma exposure increased with increasing the dose in a near-dose proportional manner. The median time of the maximum concentration (T_max_) for NAL ER occurs at 3 to 6 h post-dose, with a mean half-life (t_½_) of 6.6 to 9.6 h [3]. Its oral bioavailability is low (12−16%), with high intersubject variability (>30%) [4,5] and a high systemic clearance of 1.6 ± 0.4 L/min that approximates the liver blood flow [5].

**Figure 1:**
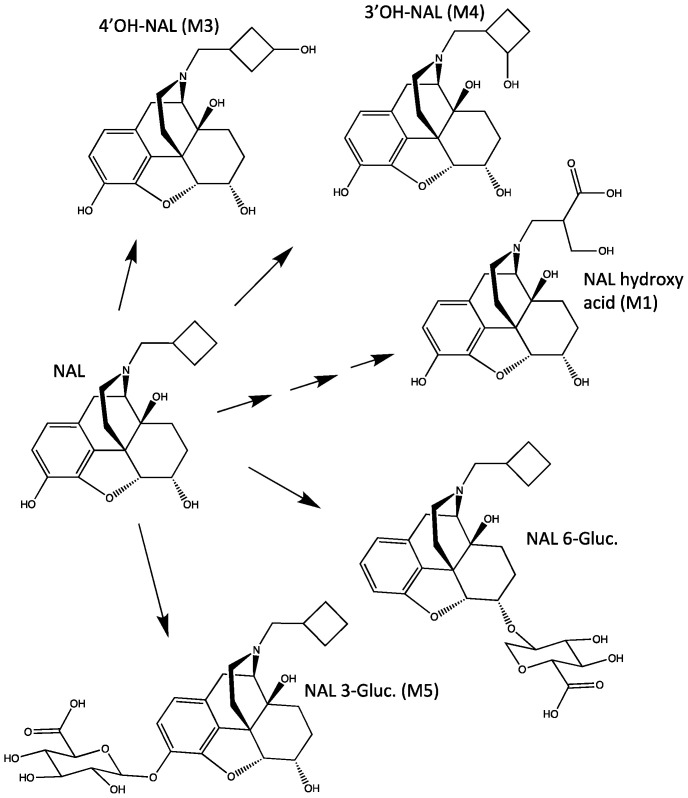
Chemical structures of NAL and its main metabolites.

NAL has low oral bioavailability, likely due to extensive first-pass metabolism. This is supported by several clinical and non-clinical studies in the literature, indicating that NAL is metabolized in the liver predominantly to hydroxylated NAL and glucuronide conjugates. Metabolites 4′-hydroxy (M3) and 3′-hydroxy (M4) nalbuphine and the 3-beta glucuronide (M5) and 6-beta glucuronide conjugates (6-Gluc-NAL) have been reported to be formed both in vitro and in vivo following the oral administration of NAL to humans [6,7] (Figure 1).

Cytochrome P450s (CYPs) 2C9 and 2C19 were previously reported to be the major enzymes for oxidative NAL metabolism. CYP2C9 is involved in the metabolism on many drugs (mostly anions) [8], and CYP2C19 has significant genetic polymorphisms [9]. Consistent with the literature, our studies confirmed M5 and M3 as the major plasma circulating metabolites (>10%), while M4 and 6-Glu-NAL were minor metabolites (<10%). In addition to these four metabolites, a previously unidentified major tri-oxidized nalbuphine metabolite, [(N-[4-hydroxy-3-methylene-butanoic acid]-nornalbuphine), M1], was isolated and successfully characterized [10].

The in vitro reaction phenotyping for NAL metabolism via human CYPs and uridine diphosphoglucuronosyltransferases (UGTs) is presented here. Considering that metabolites account for a substantial portion of NAL exposure, a dedicated hepatic impairment clinical study was conducted in subjects with mild, moderate, and severe hepatic impairment compared to healthy subjects following single oral doses of NAL ER following the FDA Guidance on Pharmacokinetics in Subjects with Impaired Hepatic Function [11]. We report herein the relationship between the extent of hepatic impairment and PK of nalbuphine and its major metabolites M5, M3, and M1. The minor metabolite M4 was also monitored, as it was suspected to be a precursor of M1. 6-Gluc-NAL was not monitored in this study.

We had previously described the development of a partial differential equation (PDE)-based model to characterize the disposition of NAL [12]. This model used a continuous intestinal model [13,14,15] interfaced to a compartmental model that included enterohepatic recycling (PDE-EHR). The recycling of NAL was modeled for both the parent drug and the glucuronide conjugate. In this model, the glucuronide was released into the intestine from the gallbladder, traversed the length of the intestine, and was converted to NAL by glucuronidase activity. The model accurately predicted the observed long half-life for oral NAL administration in healthy subjects. We have since extended the PDE-EHR model to include the disposition of the four NAL metabolites M1, M3, M4, and M5. The preliminary results of the model prediction are presented in this manuscript.

## 2. Materials and Methods

### 2.1. In Vitro Reaction Phenotyping Studies

All in vitro studies were conducted by Sekisui XenoTech, LLC (Kansas City, MO, USA). All reagents and solvents were of analytical grade. Human liver microsomes from a pool of 200 individuals (catalog number: H2610, lot number: 1210347) were used for this study. Recombinant human CYP enzymes (Bactosomes®) expressed in *Escherichia coli* and all controls were obtained from Cypex, Ltd. (Dundee, Scotland). Recombinant human UGT enzymes (Supersomes®) and the corresponding control Supersomes® (from insect cells transfected with wild-type baculovirus but no human UGT enzyme) were purchased from BD Biosciences (San Jose, CA, USA). All in vitro models were characterized internally according to the Sekisui XenoTech standard operating procedures prior to use. Test compounds nalbuphine and its deuterated standard nalbuphine-d_3_ and metabolite standards M1, M3, M4, and M5 were provided by Trevi Therapeutics. All samples were analyzed by validated LC-MS/MS multiple reaction monitoring assays.

Nalbuphine (50 µM) was incubated with recombinant human CYP enzymes (rCYP1A2, rCYP2B6, rCYP2C8, rCYP2C9, rCYP2C19, rCYP2D6, and rCYP3A4, 10 pmol CYP per incubation). Incubations were conducted at 37 ± 2 °C for 60 min. Nalbuphine was added to the incubation mixtures in 50:50 acetonitrile:water (1% *v*/*v*). Reactions were initiated by addition of the NADPH-regenerating system. The NADPH-regenerating system consisted of NADP (1 mM, pH 7.4), glucose-6-phosphate (5 mM, pH 7.4), and glucose-6-phosphate dehydrogenase (1 Unit/mL) at the final concentrations indicated. Reactions were terminated by the addition of 175 µL of stop reagent (acetonitrile containing internal standards, acetaminophen-d_4_ [300 ng/mL], and nalbuphine-d_3_ [100,000 ng/mL]). After the incubations were stopped, each sample volume was normalized to the volume of standards (0.4 mL), with 25 µL of standard blank (50:50 [*v*/*v*] acetonitrile:water). The samples were centrifuged (920× *g* for 10 min at 10 °C). The supernatant fractions were analyzed by LC-MS/MS to quantify nalbuphine, M1, M3, and M4 based on a calibration curve (0.025 to 5 µM). Zero-time samples served as 100% values to determine the percent loss of the substrate. Zero-time, zero-protein, zero-substrate, and zero-cofactor (no NADPH) samples served as blanks or negative controls.

Nalbuphine (50 µM) was manually incubated in triplicate with human liver microsomes (1 mg protein/mL) for 120 min in the presence of the chemical inhibitors (CYP1A2: 10 µM furafylline, CYP2B6: 30 µM phencyclidine, CYP2C8: 100 µM gemfibrozil glucuronide, CYP2C9: 20 µM tienilic acid, CYP2C19: 10 µM esomeprazole, CYP2D6: 5 µM paroxetine, and CYP3A4/5: 50 µM troleandomycin). Reactions were initiated and terminated as described above. Control samples with no CYP inhibitor present were conducted in the presence of the solvent used to dissolve the CYP inhibitor. Percent inhibition was calculated based on the comparison of the rate of substrate loss or metabolite formation in samples containing chemical inhibitors to the rate in the respective solvent control samples.

Nalbuphine (5 and 50 µM) was incubated with recombinant human UGT enzymes (rUGT1A1, rUGT1A3, rUGT1A4, rUGT1A6, rUGT1A8, rUGT1A9, rUGT2B4, rUGT2B7, and rUGT2B15, 0.5 mg/mL UGT). Incubations were conducted at 37 ± 2 °C for 120 min. Reactions were initiated by the addition of UDPGA and were terminated by the addition of stop reagent (acetonitrile containing the internal standards, nalbuphine-d_3_, and acetaminophen-d_4_ at 100 and 12.5 ng/mL final concentrations, respectively). Incubations of nalbuphine with microsomes from insect cells containing no human UGT enzyme served as negative controls. In addition, positive controls were included to confirm activity of the recombinant UGT enzymes (1 mM 4-methylumbelliferone for UGT1A1, 1A3, 1A6, 1A8, 1A9, 2B4, 2B7, and 2B15; 100 µM imipramine for UGT1A4). Samples were analyzed using validated LC-MS/MS assays.

### 2.2. Clinical Study Design

Study TR10 (NCT 04020016) was a phase 1, open-label, non-randomized, PK study of NAL ER in subjects with hepatic impairment (see Table S1 for details of study investigators and the Institutional Review Board). The study was conducted in subjects in the Child–Pugh categories A, B, and C, corresponding, respectively, to mild, moderate, and severe hepatic impairment—as well as age-, BMI-, and gender-matched healthy subjects as controls. Subjects with mild and moderate impairment received single ascending doses of 27 mg to 162 mg of nalbuphine tablets in a fasted state. Subjects with severe impairment received a single 27 mg dose, whereas control subjects were dosed at 162 mg only. The study was completed by 27 out of 28 enrolled subjects. Following dose administration, subjects remained in the clinic and were monitored over 5 days. The study population (n = 28) demographics are shown in Table S2. Additional study details are provided in Table S3.

### 2.3. Modeling and Simulation

**Figure 2:**
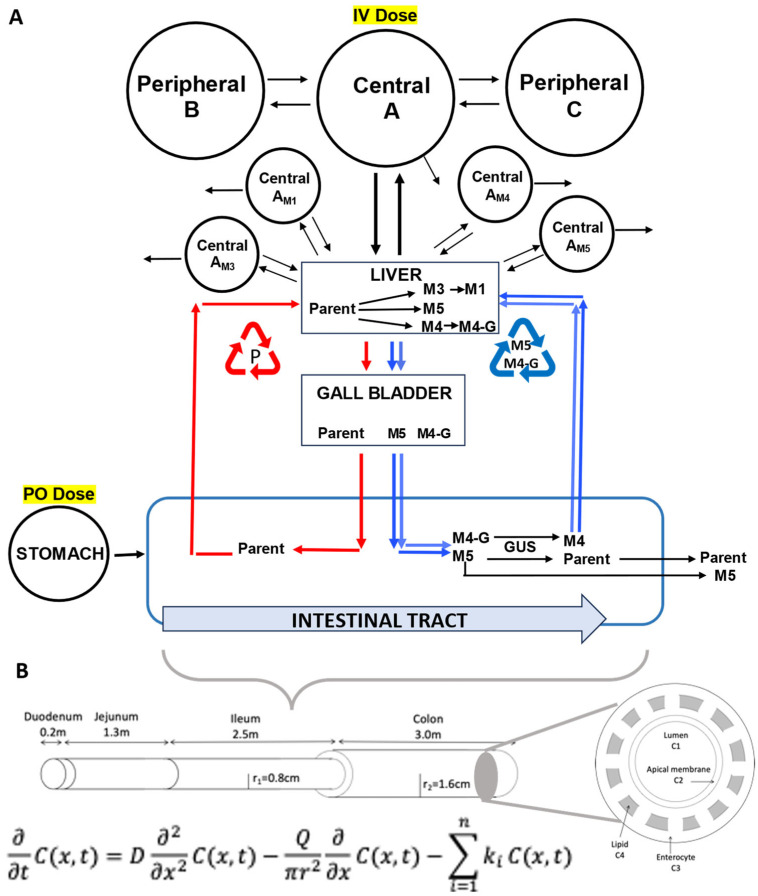
Schematic of the PDE-EHR model for the parent drug and metabolite disposition. (**A**) Upon dosing in the stomach, the parent drug moves along the length of the intestine and is absorbed into the liver compartment. Parent NAL (P) in the liver is metabolized to M3, M4, and M5. NAL, M4-G, and M5 can be secreted into the gallbladder. The gallbladder secretes parent and glucuronides into the duodenum. The parent can be recycled via absorption into the liver (red arrows). The glucuronides can be hydrolyzed by GUS expressed in the lower intestine to the parent drug, which results in recycling of the parent via glucuronidation–deglucuronidation (blue arrows). NAL and metabolites are eliminated from the system via a combination of pre-systemic loss and hepatic plus non-hepatic elimination. Open compartmental models are linked reversibly to the liver to model systemic disposition as follows: a 3-compartment model for NAL (compartments A, B, and C) and 1-compartment models for each circulating metabolite: M1, M3, M4, and M5. Sites for parent dosing (IV or PO dose) are highlighted. (**B**) Intestine is modeled as a physiological continuous compartment based on the convection–diffusion–reaction equation, with details of the cross-sectional concentric tubes depicting the lumen (drug concentration C1), apical membrane (C2), enterocyte cytosol (C3), and intracellular lipid (C4).

Modeled datasets upon NAL-ER PO dosing included plasma concentration–time profiles for NAL and its metabolites M1, M3, M4, and M5 in healthy subjects, as well as moderate and severe hepatic disease subjects. The inter-subject variability in the NAL plasma C-t profile was moderate, except in the mild group. The latter was not included in the compartment modeling due to the high intersubject variability (see the data in Figure S1). For each group, a model was developed (Figure 2) to simultaneously predict the concentration–time (C-t) profiles of NAL and all its metabolites. Since volumes of distribution (V_D_) for metabolites can be different from the parent, additional compartments for each metabolite were required. As described previously [12], these compartments have PDE dimensions but were made well stirred by including very rapid diffusion. The steps to build the overall model are presented below.

#### 2.3.1. NAL Parent Drug Modeling

Our previously reported PDE-based enterohepatic recycling model (PDE-EHR model) was parameterized on the disposition of the parent compound NAL and a first-order drug release from the NAL ER tablets [12]. The disposition of the four metabolites was incorporated into the model in a stepwise manner. First, the hepatic intrinsic clearance of NAL was partitioned into glucuronidation and oxidation. The glucuronide-mediated clearance of NAL is separate from the NAL recycled, since NAL recycling is a distribution pathway and not a clearance pathway. Hepatic clearance (CL_H_) was considered the primary clearance pathway, since minimal NAL is found as a parent in the urine. Hepatic clearance was considered to be the total clearance (CL) minus the non-hepatic clearance (CL_nH_), and the intrinsic clearance of NAL (CL_int,H_) was calculated using the well-stirred model and a blood flow of 1.45 L/min.

Due to the absence of crossover IV and PO datasets for the same population, a published IV NAL dataset [4] was used to parameterize NAL CL and a steady-state volume of distribution (V_D_) in healthy subjects, as described in detail previously [12]. Next, the observed CL/F (calculated as mean dose/AUC) from moderate or severe hepatic disease groups was compared with the healthy CL/F. For the initial simulations, the fold-change in CL/F was estimated assuming an equal change in CL (decrease) and F (increase). A factor (termed “a”) for the change in hepatic clearance between healthy and hepatic impaired subjects was calculated using CL_2_/(F_2_ a^2^) = CL_1_/(F_1_) and a = (CL_2_ F_1_/(CL_1_ F_2_))^½^, where CL_1_/F_1_ = (dose/AUC)healthy and CL_2_/F_2_ = (dose/AUC)impaired. Factor “a” was subsequently optimized with C-t profiles of NAL to decrease the hepatic clearance (CL_H_) in moderate and severe hepatic disease.

For metabolite modeling, hepatic intrinsic clearances (CL_int,H_) were modified (decreased) for moderate and severe hepatic impairment. Across all subject groups, non-hepatic elimination clearances and central volumes were held at the same value. Secretory clearances from the liver to gallbladder for NAL (CL_gi,p_) and metabolites (CL_gi,m_) were decreased as necessary for the severe impairment group.

#### 2.3.2. Metabolite M5 Modeling

The original PDE-EHR model included parent NAL being converted to the major glucuronide (M5). This model was refined to include the observed circulating metabolites from the present clinical study. The disposition of M5 was modeled as its formation in the liver from NAL, secretion into the central compartment, and recirculation via the gallbladder to the intestine, where it was de-glucuronidated and reabsorbed as a parent. Hepatic clearance of NAL consists of the M5 that is not recycled and the oxidative metabolites. M5 that is recycled is a distribution pathway. Formation and elimination clearances, as well as the percent of NAL CL_int,H_ converting to M5, were all optimized with the M5 C-t datasets. A central compartment for M5 was added (V_D_ = 15 L, approximating extracellular space). Drug recycling can greatly increase the volumes of distribution, making it difficult to estimate compartmental volumes. The central compartment was linked reversibly to the liver, where M5 was eliminated with an optimized elimination CL. The formation of CL_int_ for M5 from the parent was also optimized.

#### 2.3.3. Metabolite M3 Modeling

M3 compartments were added to the parent+M5 model. A M3 central compartment is linked reversibly to the liver, and a peripheral compartment was linked to the central compartment. Elimination occurs from the liver to form M1. Elimination clearance for M3 is modeled by both the formation of M1 and by clearance from the M3 central compartment.

#### 2.3.4. Metabolite M1 Modeling

Since M1 is a three-times oxidized metabolite of NAL (Figure 1) and M3 increases with severe hepatic impairment, a model was constructed that converted M3 to M1 in the liver. M1, a carboxylic acid alcohol, was modeled with a single compartment with an optimized volume of distribution of 53 L. Elimination of M1 occurs from the M1 central compartment.

#### 2.3.5. Metabolite M4 Modeling

M4 was modeled with enterohepatic recycling of the hydroxy metabolite. This required adding both M4 and M4-glucuronide (M4-G) central compartments. The volume of M4 was assumed to be the same as M3 and 15 L for M4-G (extracellular water). For recycling, a M4 gallbladder and M4 intestinal lumen are also required. For the healthy and moderate impairment groups, all recycling parameters (gallbladder filling and emptying and glucuronidase (GUS) activity) were identical to those published previously for parent NAL recycling.

#### 2.3.6. Other Parameters and Calculations

All non-intestinal compartments were made well stirred by adding rapid diffusion to the PDE models. Metabolite formation clearances were modified by their molecular weight ratios to keep units of mg/L. Blood to plasma ratios (BPs) are used to interconvert plasma and blood concentrations when transferring from a compartment model to the liver. All stomach and intestinal dissolution rates were identical to those reported previously for the 162 mg and 27 mg doses. Since the time after dosing for the first meal was “at least two hours” for this study and 4 h for the previously reported study [12], we use a feeding time of 3 h to model the first meal.

Experimental AUCs were calculated using the trapezoidal rule, and model AUCs were calculated by integration of the plasma concentration functions. All models were constructed in Mathematica 14.0 (Wolfram Research, Inc., Champaign, IL, USA) using the methods described previously [12].

## 3. Results

### 3.1. In Vitro Reaction Phenotyping

Table 1 and Table 2 list the results of CYP phenotyping. The recombinant human CYP enzymes involved in M3 formation were CYP2C19, CYP2C9, and CYP2C8, with minor contributions from CYP3A4 and CYP2D6. For M4 formation, the CYPs involved were CYP2C19, CYP2C9, and CYP2D6. M1 was not detected, except in one replicate of CYP2C19. Chemical CYP inhibition corroborated the major involvement of CYP2C9 and 2C19 in NAL metabolism, with multiple CYPs catalyzing the formation of M3 and M4 (Table 2).

Based on in vitro metabolism with recombinant human UGT Supersomes®, the formation of M5 was predominantly catalyzed in vitro by UGT1A8, with additional contributions from UGT2B7, UGT1A3, and UGT1A9 (Table 3).

### 3.2. Clinical Results

Clinical PK data have been presented previously at the 50th Annual Meeting of the European Society for Dermatological Research [16]. NAL C_max_ and AUC increased in a nearly dose-proportional manner in subjects with mild or moderate hepatic impairment over the 27–162 mg dose range. Mean NAL t_½_ and T_max_ were unchanged across all groups and dose levels. PK parameters for nalbuphine and its metabolites at the highest tested dose in each group are compared in Table S4.

Evaluation of NAL exposure (AUC and C_max))_ following a single dose of NAL ER 162 mg indicated the AUC and C_max_ values were similar for subjects with mild hepatic impairment as compared to healthy control subjects, whereas it was nearly three-fold and six- to eight-fold higher in subjects with moderate and severe hepatic impairment, respectively (Table S5), and no difference in the t_1/2_ or T_max_ values across all categories (Table S4).

**Figure 3:**
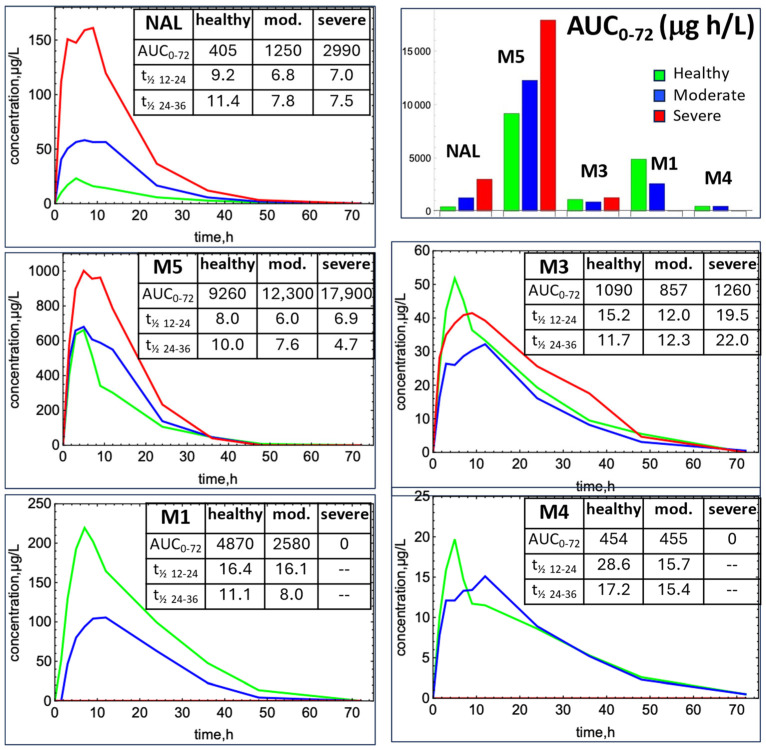
Naïve-pooled average C-t profiles following NAL administration to healthy subjects (green), subjects with moderate hepatic impairment (blue), and severe hepatic impairment (red). Severe impairment data were dose-normalized to the healthy and moderate impairment groups. Concentrations of metabolites M1 and M4 were below the limit of quantitation for the severe impairment subjects. The AUC and t_½_ calculations are based on average C-t profiles of naive-pooled data. The AUCs are in μg h/L and t_½_ in h. Interindividual variabilities for each mean are shown in Figure 4.

The naïve-pooled average C-t profiles for modeling (healthy, moderate, and severe hepatic impairment) are shown in Figure 3. For the severe group (27 mg dose), the concentrations are dose-normalized to the healthy and moderate impairment dose (162 mg). In the healthy group, the most abundant metabolites are M5 > M1 >> M3 > M4. NAL exposure increased dramatically with moderate and severe hepatic impairment, with the dose-normalized AUCs increasing by 3.1- and 7.4-fold, respectively. The AUCs for M5 also increased by 1.3- and 1.9-fold with moderate and severe hepatic impairment, respectively. M3 decreased 1.3-fold for moderate impairment, then increased 1.2-fold for severe impairment. M1 decreased 1.9-fold for moderate impairment and was below the lower limit of quantitation (LLOQ) for the severe group. The M4 AUC did not change for the moderate group and was below the LLOQ for the severe group.

### 3.3. Modeling

The optimized parameters for all groups are shown in Table 4. The initial values of factor “a”, assuming equal changes in CL and F with hepatic disease, were 1.8 and 2.5 for the moderate and severe impairment groups, respectively. The final factor “a” optimized with the observed data was 1.4 and 1.7 for the moderate and severe impairment groups, respectively. In the final optimized model for healthy subjects, 40% NAL was modeled as converting to M5 in healthy livers, 55% in moderate hepatic disease, and 60% in severe hepatic disease. For the control group, M3 formation was optimized to 50% of the hepatic intrinsic clearance of NAL. The BP values were 1.15 for NAL (measured); 0.56 for M5 (no erythrocyte partitioning); and 0.9, 0.9, and 0.7, respectively, for M3, M4, and M1. Based on the observed C-t profile, the volume of distribution for M3 was optimized to 214 L. The M4 volume was assumed to be identical to M3, and the volume of distribution for M1 was optimized to 53 L. It is important to note that, since the BP values for the NAL metabolites were not experimentally determined, the estimated BP values were empirically established for the purpose of this preliminary modeling. Since the BP will be correlated with volumes [17,18], both parameters cannot be optimized simultaneously. While the BP values may not be accurate, they are consistent with the expected volume changes, since V_D_ is primarily determined by membrane partitioning and plasma protein binding [19,20].

**Figure 4:**
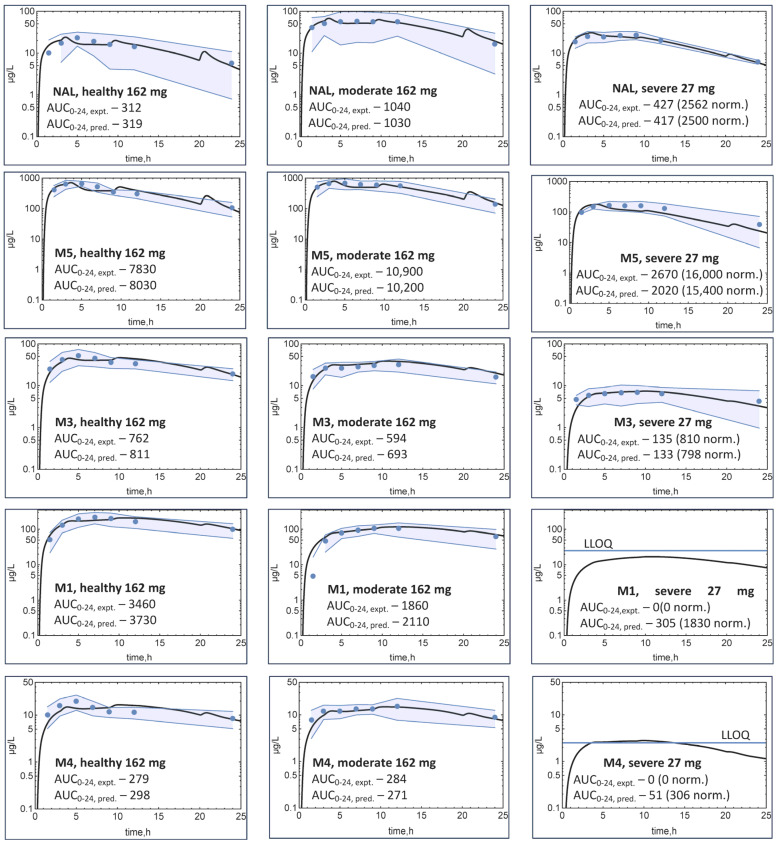
Predicted NAL and metabolite plasma concentration–time profiles based on the PDE-EHR model following the administration of a NAL ER 162 mg oral dose to healthy subjects, subjects with moderate hepatic impairment, and a NAL ER 27 mg dose in subjects with severe hepatic impairment. Severe impairment C-t profiles were not dose normalized to the healthy and moderate impairment groups. Circles represent average experimental data, and the black solid line is the model prediction. Standard deviation of the clinical data is depicted with the gray-shaded areas. Experimental and predicted AUC_0–24_ values are listed.

For the moderate and severe impairment groups, all hepatic formation and elimination clearances decreased for the NAL and metabolites. As seen previously with crossover data, NAL disposition in healthy subjects was reproduced with the PDE-EHR model (Figure 4). The addition of the M5, M3, M1, and M4 components resulted in predicted C-t profiles generally within the observed clinical variability (gray-shaded areas in Figure 4). The average absolute percent error for all predicted AUCs is 7%.

## 4. Discussion

The goal of this study was to evaluate the ability of the PDE-EHR model to model metabolite formation for NAL in healthy and hepatic impaired subjects following oral administration. We are encouraged that all main metabolites of NAL in the healthy subjects could be modeled by adding simple compartmental components to the original PDE-EHR model. For the moderate and severe hepatic impairment subjects, the C-t profiles could be simulated by decreasing all hepatic intrinsic terms while leaving all non-hepatic elimination clearances constant. Even the unusual behavior of M3, decreasing exposure for moderate impairment and increasing for severe impairment, can be reproduced by assuming that M1 is a product of the further oxidation of M3.

Other important observations from the clinical data are that hepatic impairment decreased CYP-mediated metabolite exposure to a greater extent than glucuronide formation. The data in Table 4 suggest that F and CL are not equally impacted by hepatic impairment (initial versus optimized “a” values). IV data would be needed to determine the F and CL. Also, it appears that all intrinsic formation clearances decrease substantially, with healthy to severe decreases of 92%, 88%, and 80% for M3, M4, and M5, respectively (Table 4). M5 exposure increased with hepatic disease, despite the decrease in its formation clearance. This observation is explained by the model-predicted mass balance shift of NAL formation to M5 relative to M3. M3 and M5 are the two major primary metabolites, and a relatively greater decrease in M3 formation clearance shifts NAL conversion to M5, thereby predicting an increased M5 exposure. The decrease in both CYP and UGT activity with hepatic impairment is consistent with reports of message and protein expression [21,22].

A recent report on the impact of hepatic impairment on NAL after IV administration did not show a significant effect on clearance for a moderate/severe hepatic impairment group [23]. Instead of a decrease in clearance, a small (statistically non-significant) increase in clearance was observed. This difference could be due to some combination of differences in disease classification (Child–Pugh versus NCI-ODWG) [23], number of subjects, subject demographics, or population genotype.

Changes in M3 exposure with hepatic impairment are complex presumably due to secondary hepatic metabolism to M1. The observed M3 t_½_ between 24 and 36 h increased from 12 h for healthy to 22 h for severe impairment. This observation was successfully modeled as there was a decreased hepatic clearance of M3 to form M1. Also, the greater impact of hepatic impairment on the conversion of M3 to M1 (possibly, two additional hepatic oxidations) is likely the reason M3 AUC decreased for moderate hepatic impairment then increased for severe impairment. This was also reproduced with the PDE-EHR model.

While both M3 and M4 could possibly be precursors of M1, most of the M1 C-t profile could be reproduced assuming that M3 is the precursor. However, the M1 metabolite shows a significant lag time (Figure 4, M1, moderate), with most subjects having M1 levels below the limit of quantitation (BLQ) at 1.5 h. This lag time was not reproduced by the model. The need for three oxidation steps to form M1 is the likely origin of this lag time, but the mechanism of M1 formation is unknown.

The clinical data in healthy subjects show similar t_½_ between 24 and 36 h for NAL, M3, and M5, suggesting formation-limited clearance for these two metabolites (Figure 3). M4 has longer t_½_ values for both 12–24 h and 24–36 h, possibly due to recycling of a hydroxy glucuronide metabolite. This assumption is reasonable, since hydroxy glucuronide metabolites have been detected in the plasma of hemodialysis subjects and urine of healthy subjects (internal data). Therefore, we added recycling pathways for M4. The observed M4 C-t profile could be reproduced using the same gallbladder input and output clearances and the same intestinal glucuronidase function as used for NAL.

The in vitro data presented in Table 1 and Table 2 show that NAL oxidation is mediated primarily by CYP2C9 and 2C19, with minor contributions from other CYPs. All CYPs involved have been shown to have decreased activity with hepatic impairment consistent with the modeling results. The UGT data in Table 3 suggest that NAL can be conjugated by several UGTs, with expressed UGT1A8 having the highest activity. UGT1A8 involvement has not been previously reported, most likely because it was not included in the in vitro studies [7]. Although UGT1A8 has been reported to be primarily expressed in intestinal tissue [24,25], it has been observed in hepatocytes as well [26]. Currently, UGT-specific inhibitors are not available, and expressed enzyme activity cannot be used for quantitative phenotyping. However, since severe hepatic impairment results in a seven-fold increase in NAL AUC (Table S4 and Figure 3) and the glucuronides remain the major excreted metabolite [7], it is likely that the hepatic UGTs play a larger role in NAL elimination than intestinal UGTs [12]. Additional phenotyping data will be necessary to characterize this metabolic pathway.

There are caveats in this modeling effort, and several assumptions were necessary. Most importantly, these efforts are based on assessing the hepatic impairment following oral dosing only, and no IV data are available in the same subjects. We have previously shown that, in the absence of a crossover IV/oral study, the modeling results were similar for NAL disposition whether we used internal cross-over data or published IV datasets from different subjects [12]. For this study, we used the IV data reported by Aitkenhead [4].

In the absence of PK data following dosing of metabolite, neither their clearance nor V_D_ parameters is known. Instead, we assumed that all metabolite elimination clearances were not changed for hepatic impairment relative to healthy subjects, and assumed that the glucuronide metabolites have V_D_ values of 15 L (extracellular space). Relative to NAL V_D,_ the optimized hydroxy metabolite V_D_ values decreased by 20%, and M1 (a hydroxy carboxylic acid) decreased by 80%. Decreases in V_D_ are expected due to the hydrophilic nature of these metabolites and are expected to be correlated with metabolite elimination clearances and their assumed BP values.

In healthy subjects, metabolites formed in the gut that are not recycled contribute minimally to first pass metabolism [12]. Recycled metabolites are part of drug distribution, since the drug will reappear in the plasma eventually. Since IV dosing in hepatic impaired subjects was not conducted, we were unable to determine the degree of intestinal metabolism in these subjects. Even though severe hepatic impairment results in a 7-fold increase in AUC (Table S4), gut metabolism cannot be entirely excluded in hepatic impaired subjects considering that most NAL-metabolizing enzymes, namely CYP3A4/5 and UGTs 1A8, 1A9, and 2B7, are also expressed in the intestines [27]. CYP3A4/5 oxidation, although a minor contributor to NAL elimination, can decrease in both the liver and the gut with hepatic impairment [28]. The PDE-EHR model, optimized to the observed datasets, predicts a minimal impact of the gut metabolism. Since CYP3A4/5 is a minor contributor to NAL oxidative elimination (17%, Table 2), drug interactions due to CYP3A4/5 inhibition are not expected in healthy subjects. Also, given the contribution of CYP2C19 to NAL oxidative metabolism (29%, Table 2), and the observation that major NAL metabolism is via glucuronidation, CYP2C19 polymorphisms are unlikely to significantly impact NAL pharmacokinetics.

Some of the numerous pathophysiological changes in hepatic disease that will alter the drug disposition remain to be explored with the PDE-EHR model. For example, plasma protein binding will likely decrease. However, the impact on clearance will likely be minimal, since NAL has a low protein binding (~50% [3]) with a high extraction ratio (ER) drug in healthy subjects and moderate ER drug in severe impairment subjects (ER = 0.82 and 0.47, respectively) calculated from intrinsic clearance values in Table 4. The volume of distribution could be affected, but better estimates of volumes of distribution will be required for meaningful simulations. The impact of decreased plasma protein binding has been discussed previously [28,29]. Alterations in the liver blood flow in hepatic impairment can also be incorporated in modeling [28,30].

The structure of M1 was determined by synthesis and NMR characterization [10], but the metabolic pathways for the formation of M1 are currently unknown. Since CYPs 2C9 and 2C19 oxidize the cyclobutyl ring at the 3′-(M4) and 4′-(M3) positions, these are likely steps for the formation of M1. Ring opening reactions have been reported for CYPs, and the proposed mechanisms include Bayer–Villager-type oxidation to form lactones [31,32] or other peroxy intermediate mediated reactions [33,34]. Another possibility is that M3 is oxidized to the ketone followed by lactone formation with FMO5 [35]. A lactone could be a precursor to the hydroxy acid (M1), since interconversion between hydroxy acids and lactones can occur in vivo [36]. However, the lactone was not detected in the plasma samples. Efforts to identify the metabolic pathways to M1 are currently in progress.

**Figure 5:**
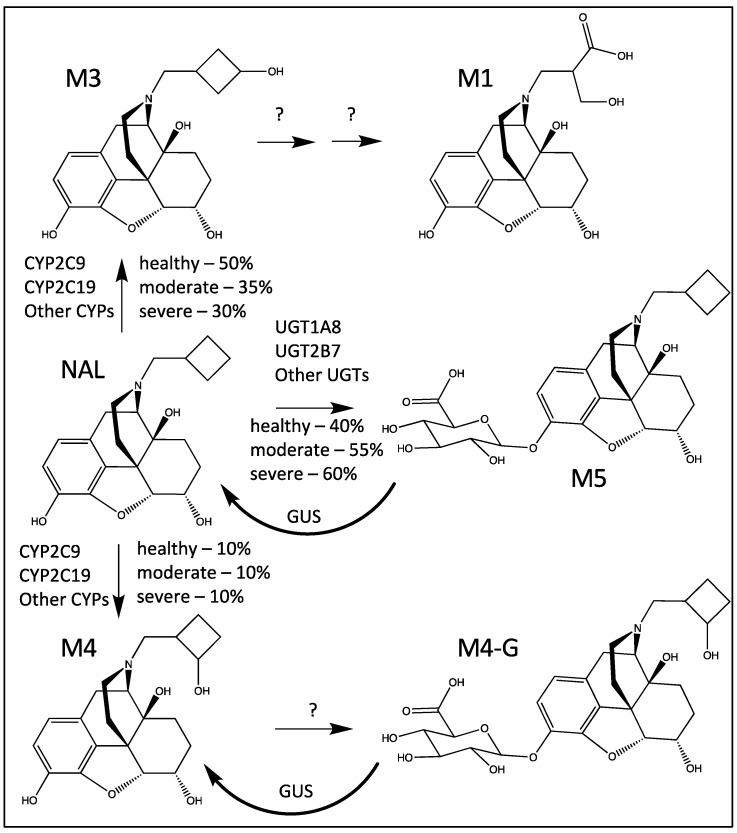
Proposed metabolic pathways and enzymes for NAL metabolism in healthy moderate and severe hepatic impairment. The percent of NAL intrinsic clearance for each primary metabolite (M3, M5, and M4) were optimized to reproduce the C-t profiles of NAL and all main metabolites. The nalbuphine 6-gluc is also formed but its structure is not shown in this figure.

The proposed metabolic pathways and enzymes for NAL metabolism are shown in Figure 5. The PDE-EHR model can predict the complex disposition of NAL, as well as its metabolites, in both healthy and hepatic disease groups (Figure 4). Mechanistic information from in vitro reaction phenotyping assays can be easily incorporated into the model. The model has additionally aided in providing insight into potential pathways of M1 formation. We expect the model to be extremely useful for predictions of complex drug disposition in other hepato-gastric disease conditions. Another key utility of the model is in predicting drug–drug interactions for a drug of interest, either as a victim or a perpetrator. The PDE-EHR model has the potential to improve drug safety predictions, as well as drug efficacy predictions in combination with pharmacodynamic modeling. With additional application to other drugs and refinement of this model in future studies, we expect to develop a robust mechanistic modeling framework to significantly improve the predictions of complex drug disposition.

The model and results presented here should be considered as preliminary. Additional in vitro data and predicted V_D_ of the metabolites can remove some of the correlation between the parameters (BP, V_D_, and CL). The C-t profiles for the NAL metabolites provide a rich dataset for parameter estimation and may be useful for future studies, e.g., NAL–drug interactions, special populations, etc. Whereas the continuous absorption model appears to be useful for a variety of drugs [13,14,37], incorporating enterohepatic recycling has only been attempted for NAL. The application of this model to other drugs will depend on several factors. C-t profiles and in vitro data for parent drug and all metabolites are necessary, since a model must fit within the constraints of the observed C-t profiles and must remain consistent with mechanistic in vitro data. As with any model, validation requires successful predictions across a variety of drug molecules. NAL is a BCS Class I drug, with no transporter-mediated disposition. Although the continuous absorption model used here can be easily modified to include complexities such as solubility, precipitation, intestinal metabolism, transport, and enterohepatic recycling, further studies with other drugs are necessary.

## 5. Conclusions

In summary, the complex pharmacokinetics of NAL and its metabolites were well predicted in healthy subjects and moderate/severe hepatic disease by the PDE-EHR model. The model corroborates the in vitro reaction phenotyping results and provides insights into potential mechanisms of formation of a major metabolite of NAL, M1. Future work with the PDE-EHR model includes the prediction of drug–drug interactions and drug safety.

## Figures and Tables

**Table 1:** CYP reaction phenotyping for the NAL disappearance and appearance of oxidative metabolites M1, M3, and M4. NAL (50 µM) was incubated for 60 min with recombinant human CYP enzymes (10 pmol CYP per incubation). Mean values of triplicate experiments are listed.

Test System	NAL Loss (%)	M1 (pmol)	M3 (pmol)	M4 (pmol)
Control Bactosomes^®^	No loss	ND ^1^	ND	ND
Reductase Control	3.3	ND	ND	ND
CYP1A2	1.6	ND	ND	ND
CYP2B6 + b5	No loss	ND	ND	ND
CYP2C8 + b5	3.6	ND	16.6	ND
CYP2C9 + b5	4.0	ND	305	51.0
CYP2C19 + b5	27	1.81 ^2^	1390	93.2
CYP2D6	No loss	ND	5.95	31.5
CYP3A4 + b5	10.3	ND	6.01	ND

^1^ ND: not detected. ^2^ Value is the mean of triplicate determinations; however, only one replicate contained a peak at a level exceeding the lower limit of quantification for the analytical method.

**Table 2:** Metabolism-dependent chemical inhibition in HLM (1 mg protein/mL), with 50 µM NAL at 120 min, with a 30-min pre-incubation time.

			% Inhibition (SD ^1^)	
Targeted CYP	Inhibitor (μM)	NAL	M3	M4
CYP1A2	Furafylline (10)	None	14.6 (0.4)	7.5 (0.4)
CYP2B6	Phencyclidine (30)	None	None	18.3 (0.4)
CYP2C8	Gemfibrozil glucuronide (100)	None	0.6 (0.03)	20.3 (0.6)
CYP2C9	Tienilic acid (20)	34.6 (1.6)	60.8 (4.9)	84.1 (1.6)
CYP2C19	Esomeprazole (10)	28.7 (1.4)	22.5 (1.7)	24.6 (1.6)
CYP2D6	Paroxetine (5)	None	7.9 (1.6)	22.9 (0.5)
CYP3A4/5	Troleandomycin (50)	17.0 (0.5)	4.2 (ND ^2^)	19.0 (1.2)

^1^ SD: Standard deviation. ^2^ ND: not determined due to lack of replicates.

**Table 3:** UGT reaction phenotyping for NAL disappearance and appearance of glucuronidated metabolite M5. NAL (5 µM) was incubated for 120 min with recombinant human UGT Supersomes^®^ (0.5 mg protein/mL). Mean values of triplicate experiments are listed.

Test System	NAL Loss (%)	M5 (pmol)
Control Supersomes	3.9	ND ^1^
UGT1A1	3.4	ND
UGT1A3	22.5	162
UGT1A4	3.7	ND
UGT1A6	2.9	ND
UGT1A8	69.9	621
UGT1A9	15.0	84.8
UGT2B4	2.4	ND
UGT2B7	26.0	169
UGT2B15	1.7	ND

^1^ ND: not detected.

**Table 4:** Model inputs and pharmacokinetic parameter estimates used in the optimized model.

Parameter (Units)	Healthy	Moderate	Severe
CL_gi,p_ = Cl_gi,m_	0.25 CL_int,H_	0.25 CL_int,H_	0.1 CL_int,H_
Observed CL/F (L/h)	465	142	58
V_D_ (L) simulated	267	267	267
Initial factor “a” for change in CL and F ^1^	NA ^2^	1.8	2.5
Optimized “a”	NA ^2^	1.4	1.7
CL_H_ (L/h)	78.8	51.5	32.4
CL_nH_ (L/h)	4.14	4.14	4.14
CL_int,H_(L/h)	322	92	42
Fraction of NAL CL_int_ forming M3	0.5	0.35	0.3
Fraction of NAL CL_int_ forming M4	0.1	0.1	0.1
Fraction of NAL CL_int_ forming M5	0.4	0.55	0.6
Formation CL_int_ for M1 (L/h)	60	40	30
Formation CL_int_ for M3 (L/h)	161	32.3	12.5
Formation CL_int_ for M4 (L/h)	32.2	9.2	4.2
Formation CL_int_ for M4-G (L/h)	5	5	5
Formation CL_int_ for M5 (L/h)	129	51	25
M1 V_D_ (L)	53	53	53
M3 V_D_ (L)	214	214	214
M4 V_D_ (L)	214	214	214
M5 V_D_ (L)	15	15	15
Elimination CL for M1 (L/h)	20	20	20
Elimination CL for M3 (L/h)	4	4	4
Elimination CL for M4 (L/h)	40	40	40
Elimination CL for M5 (L/h)	17	17	17

^1^ Calculated as the square root of fold-change in CL/F between the subject and healthy group. ^2^ Not applicable.

## Data Availability

The code and all data used for modeling are included in the Supplementary Information.

## Supplementary Materials

The following supporting information can be downloaded at https://www.mdpi.com/article/10.3390/metabo14090471/s1: Table S1. Study investigators and IRB details; Table S2. Summary of the demographic characteristics of each hepatic impairment function group; Table S3. Details of the clinical study design, sample collection, and analysis; Table S4. Mean (SD) nalbuphine and its metabolites M1, M3, M4, and M5 plasma PK parameters and exposure ratios (hepatic impaired subjects/healthy control subjects) following oral administration of nalbuphine extended release to healthy and hepatic impaired subjects; Table S5. Oral dosing PK metrics of nalbuphine in hepatic impaired subjects relative to healthy (control) matched subjects; Figure S1. Concentration–time plots for nalbuphine in healthy and hepatic impairment groups, and Mathematica code for the PDE-EHR model, with moderate group datasets as example input.
