# Supplementary material for: Disposition of Oral Nalbuphine and Its Metabolites in Healthy Subjects and Subjects with Hepatic Impairment: Preliminary Modeling Results Using a Continuous Intestinal Absorption Model with Enterohepatic Recirculation
Source: Metabolites. 2024 Aug 27;14(9):471. doi: 10.3390/metabo14090471 (PMC11433732; doi:10.3390/metabo14090471)
Supplement: Supplementary file 1 [file metabolites-14-00471-s001.zip › metabolites-3152146-supplementary.pdf]

Disposition of Nalbuphine and its Metabolites in Healthy Subjects and Subjects with Hepatic Disease: Preliminary Modeling Results Using a Continuous Intestinal Absorption Model with Enterohepatic Recirculation

Supplementary Materials

Supplementary materials are organized as follows:

I] Details of clinical study

Table S1. Study investigators and IRB details

Table S2. Summary of demographic characteristics of each hepatic impairment function group

Table S3. Details of clinical study design, sample collection and analysis

Table S4: Mean (SD) nalbuphine and its metabolites NAL-Acid (M1), 4OH-NAL (M3), 3-OH NAL (M4) and 3-Glu-NAL (M5) plasma PK parameters and exposure ratios (hepatic impaired subjects/healthy control subjects) following oral administration of nalbuphine extended-release to healthy and hepatic impaired subjects

Table S5. Oral dosing PK metrics of nalbuphine in hepatic impaired subjects relative to healthy (control) matched subjects

Figure S1: Concentration-time plots for nalbuphine in healthy and hepatic impairment groups

II] Mathematica code for the PDE-EHR model, with moderate group datasets as example input.

I] Details of clinical studies

Ethical Conduct of the Study

This investigation was carried out in accordance with the basic ethical principles put forth in the Declaration of Helsinki of the World Medical Assembly and its revisions (with the exception that registration of such Phase 1 trials in a publicly accessible database is not mandatory), as well as the rules of Good Clinical Practice (GCP) of the US FDA (Protection of Human Subjects, 21 CFR 50; IRB, 21 CFR 56; and IND, 21 CFR 312.

List of Investigators and IRB

The Investigators for the study obtained Institutional Review Board (IRB) approval for the protocol, all protocol amendments, and the written informed consent prior to study initiation, in conformance with 21Code of Federal Regulations (CFR) 56. Information regarding the IRB approval is listed in table S1 below.

Table S1. Study Investigators and IRB Details

Site	Location	Investigator	IEC/IRB
Division of Clinical Pharmacology, University of Miami	Miami, Florida 33136 USA	Richard A. Preston MD, MSPH, MBA	**Independent ethics committee)** **Advarra** Columbia, Maryland 21046<br>FDA/OHRP IRB Registration Number: IRB00000971<br>Independent Investigational<br><br>www.advarra.com
inVentiv Health Clinical Research Services	Miami, Florida 33136 USA	David J. Wyatt MD	
Orlando Clinical Research Center, Inc.	Orlando, FL 32809 USA	Thomas C. Marbury, MD	

Subject Information and Consent

Informed consent from each subject participating in this study was obtained prior to dosing. The document was in a language understandable to the subject. The consent was obtained in accordance with 21 CFR 50. Likewise, compliance with the Health Insurance Portability and Accountability Act (HIPAA) was ensured. Every effort was made to maintain anonymity and confidentiality of medical records during this investigation.

**Table S2.**	**Summary of Demographic Characteristics of Each Hepatic Impairment Function Group**			
**Child-Pugh Category**	**Healthy (Normal)**	**Mild (CP-A)**	**Moderate (CP-B)**	**Severe (CP-C)**
**Total Grade Score**	**N/A**	**5 to 6 points**	**7 to 9 points**	**10 to 15 points**
**Number of subjects**	**8**	**8**	**8**	**4**
Age (years)				
Mean	55.9	59.5	60.5	60.3
SD	5.7	4.9	5.7	9.0
Median	53.5	60.0	61.5	61.0
Min, Max	50, 67	49, 64	54, 69	51, 68
Sex				
Female (%)	3 (37.5)	4 (50.0)	1 (12.5)	2 (50.0)
Male (%)	5 (62.5)	4 (50.0)	7 (87.5)	2 (50.0)
Ethnicity				
Hispanic or Latino (%)	8 (100)	1 (12.5)	1 (12.5)	1 (25.0)
Not Hispanic or Latino (%)	0	7 (87.5)	7 (87.5)	3 (75.0)
Race				
White (%)	6 (75.0)	4 (50.0)	6 (75.0)	3 (75.0)
Black (%)	2 (25.0)	3 (37.5)	2 (25.0)	1 (25.0)
Asian (%)	0	1 (12.5)	0	0
Height (cm) n	8	8	8	4
Mean	163.88	166.94	173.85	166.75
SD	9.17	8.09	7.45	8.97
Median	164.00	168.25	175.55	163.00
Min, Max	146.0, 174.0	152.2, 176.4	159.8, 182.1	161.0, 180.0
Weight (kg) n	8	8	8	4
Mean	80.33	79.64	95.99	83.63
SD	8.40	16.21	15.02	15.60
Median	77.90	75.05	99.10	79.45
Min, Max	71.1, 92.8	63.0, 103.2	76.6, 114.7	70.6, 105.0
BMI (kg/m^2) n	7	8	7	8
Mean	29.963	28.650	31.638	29.9000
SD	2.566	5.800	3.559	3.295
Median	29.700	27.300	31.100	30.300
Min, Max	26.80, 34.70	20.20, 36.40	27.00, 37.10	26.10, 32.90
Abbreviations: BMI = body mass index; Max = maximum; Min = minimum; N = number of subjects; n = number of subjects with data available for given category; SD = standard deviation.				

<table>
<tr><td colspan="2">Table S3. Details of Clinical Study Design, Sample Collection and Analysis (NCT04020016)</td></tr>
<tr><td>Clinical Study (Completion Date)</td><td>16 May 2021</td></tr>
<tr><td>Brief Description</td><td>Single dose PK in in subjects with impaired hepatic Function (Child-Pough [CP] category A (CP-A, mild), CP-B (moderate) and CP-C (severe) compared to healthy subjects in the fasted state over a 27, 54, 108, 162 mg dose range (approx. 30, 60, 120, and 180 mg nalbuphine HCl, respectively).</td></tr>
<tr><td>Nalbuphine Treatment</td><td>Single dose administered as single NAL ER tablets in fasted state
<table>
<tr><th>NAL Free base dose</th><th>Nominal NAL HCl salt</th><th>Subject Group</th></tr>
<tr><td>1x 27 mg</td><td>30 mg</td><td>CP-A; CP-B, CP-C</td></tr>
<tr><td>1 x 54 mg</td><td>60 mg</td><td>CP-A; CP-B only</td></tr>
<tr><td>1 x 108 mg</td><td>120 mg</td><td>CP-A; CP-B only</td></tr>
<tr><td>1x 162 mg</td><td>180 mg</td><td>CP-A; CP-B and Healthy</td></tr>
</table></td></tr>
<tr><td>Study Design</td><td>Phase 1, open-label, non-randomized, parallel-groups</td></tr>
<tr><td>Dose administration</td><td><u>Fasted State:</u>

Subjects were fasted overnight for at least 8 hours prior to dosing and at least 2 hours post-dosing.
• Nalbuphine tablet was administered orally with 240 mL
• Water, soft drinks allowed beginning 1 hour after dosing
• No food allowed for at least 2 hours post-dose
• Standard breakfast consumed at approximately 2 h after dose</td></tr>
<tr><td>PK Blood Sampling (time)</td><td>Pre-dose (0) and 1.5, 3, 5, 7, 9, 12, 24, 36, 48, and 72 hours post-dose</td></tr>
<tr><td>Plasma Bioanalysis</td><td>Nalbuphine and its metabolites were assayed using a validated LC-MS/MS method conducted at Covance Labs, Madison WI</td></tr>
<tr><td>PK Analysis</td><td>PK analysis was performed using Phoenix WinNonlin® version 8.0 or higher, safety data tables and listings, as well as PK tables and listings were created using SAS®, release 9.2 or a higher version.</td></tr>
</table>

**Table S4. Mean (SD) Nabuphine and its Metabolites M1, M3, M4 and M5 Plasma PK Parameters Following Oral Administration of Nalbuphine Extended-Release to Healthy and Hepatic-Impaired Subjects**								
**Child-Pugh Category**	**Healthy (Normal)**		**Mild (CP-A)**		**Moderate (CP-B)**		**Severe (CP-C)**	
**Dose (mg)**	**162 mg**		**162 mg**		**162 mg**		**27 mg**	
**Parameter (Units)**	**Mean (N=8)**	**SD**	**Mean (N=7)**	**SD**	**Mean (N= 6)**	**SD**	**Mean (N=4)**	**SD**
**NALBUPHINE**								
$AUC_{(0\text{-last})}$ (ng·hr/mL)	417.8	293.3	375.27	118.5	1234.0	784.1	489.2	98.5
C_{max} (ng/mL)	27.0	9.2	19.9	7	65.4	36.2	28.3	6.2
T_{max} (h)	5.6	1.9	9.8	7.2	7.2	3.1	6.0	3.5
$t_{1/2}$ (hr)	9.9	2.5	9.5	1.7	8.7	2.8	7.2	0.9
**NAL-Acid Metabolite M1**								
$AUC_{0\text{-}t}$ (h*ng/mL)	4331.9	1937.8	5225.1	3409.8	2348.5	1233.9	BLQ	--
C_{max} (ng/mL)	216.0	86.3	223.0	125.0	115.0	43.4	BLQ	--
T_{max} (h)	7.3	0.8	11.8	5.7	12.2	6.1	--	--
$T_{1/2\ el}$ (h)	13.6	5.2	11.5^	2.4	11.9*	1.0	--	--
**4OH-NAL Metabolite M3**								
$AUC_{0\text{-}t}$ (h*ng/mL)	1045.6	301.0	1023.3	319.2	813.2	177.8	186.1	122.4
C_{max} (ng/mL)	55.8	20.7	41.2	13.8	35.8	10.5	7.8	3.0
T_{max} (h)	5.7	2.9	9.4	7.4	7.5	3.2	5.9	4.7
$T_{1/2\ el}$ (h)	13.7	2.6	13.1	1.6	13.0	2.9	26.12*	5.6
**3OH-NAL Metabolite M4**								
$AUC_{0\text{-}t}$ (h*ng/mL)	426.4	193.9	412.2	180.6	418.4	179.9	30.9*	6.9
C_{max} (ng/mL)	22.3	7.3	15.3	6.4	16.8	7.0	4.1*	0.1
T_{max} (h)	4.4	1.0	9.7	7.2	7.5	3.2	5.0	2.8
$T_{1/2\ el}$ (h)	18.7	4.5	23.0	4.4	17.8	3.9	--	
**3-Glu-NAL Metabolite M5**								
$AUC_{0\text{-}t}$ (h*ng/mL)	8952.7	2304.6	12451.6	4485.5	11973.2	3492.8	2205.9	1251.9
C_{max} (ng/mL)	755.0	168.0	743.0	243.0	810.0	159.0	185.0	51.0
T_{max} (h)	4.1	1.6	5.9	8.1	6.2	3.6	7.3	4.0
$T_{1/2\ el}$ (h)	8.7	2.7	9.2	2.2	6.8	1.9	5.5^^	--

-- Could not be calculated ^N=4, .*N = 2; n=1^^ BLQ: below the limit of quantitation

**Table S5. Oral Dosing PK Metrics of Nalbuphine in Hepatic Impaired Subjects Relative to Healthy (Control) Matched Subjects**						
**Parameter (Units)**	**Adjusted gMean**		**Ratio[1]**	**90% CI[2] (%)**	**%CV**	**P-Value**
	**Mild (Child Pugh A) (N=7)**	**Matched Healthy (n=7)**				
$AUC_{0\text{-}t}$ (h*ng/mL)	357.3	355.4	100.5	63.8 - 158.5	50.6	0.984
$AUC_{0\text{-}inf}$ (h*ng/mL)	356.1	374.8	95.0	58.4 - 154.6	51.8	0.854
C_{max} (ng/mL)	18.7	25.7	72.9	51.4 - 103.3	37.9	0.132
**Parameter (Units)**	**Moderate (Child Pugh B) (n=7)**	**Matched Healthy (n=7)**	**Ratio[1]**	**90% CI[2] (%)**	**%CV**	**P-Value**
$AUC_{0\text{-}t}$ (h*ng/mL)	1050.6	355.4	295.6	162.3 -538.4	65.8	0.008
$AUC_{0\text{-}inf}$ (h*ng/mL)	1074.1	374.8	286.6	159.4 -515.2	64.1	0.008
C_{max} (ng/mL)	57.2	25.7	222.6	139.8 -354.5	49.2	0.010
**Parameter (Units)**	**Severe (Child Pugh C) (n=4)^**	**Matched Healthy (n=7) ^**	**Ratio[1]**	**90% CI[2] (%)**	**%CV**	**P-Value**
$AUC_{0\text{-}t}$ (h*ng/mL)	482.3	59.2	814.2	466.5 - 1421.0	51.5	<0.0001
$AUC_{0\text{-}inf}$ (h*ng/mL)	491.3	62.5	786.4	454.1 - 1361.9	50.7	<0.0001
C_{max} (ng/mL)	27.8	4.3	647.7	455.4 - 921.1	31.4	<0.0001

AUC, area under concentration–time curve in plasma; $AUC_{0\text{–}\infty}$, AUC from time 0 extrapolated to infinity; $AUC_{0\text{–}tz}$, AUC from time 0 to last quantifiable plasma concentration; C_{max}, maximum measured concentration in plasma; CI, confidence interval; gMean, geometric least square mean.

^NAL Dose: 27 mg. Control subjects (162 mg) dose-normalized to 27 mg.

Probability (p) values derived from Type III sums of squares.

[1] Calculated using least-squares means according to the formula: exp (DIFFERENCE) * 100.

[2] 90% Confidence Interval calculated according to the formula: exp (DIFFERENCE ± $t_{(dfResidual)}$* $SE_{DIFFERENCE}$).

[3] Intersubject variability (CV) Calculated according to formula: 100 * SQRT ($e^{[MSE]}$ - 1)

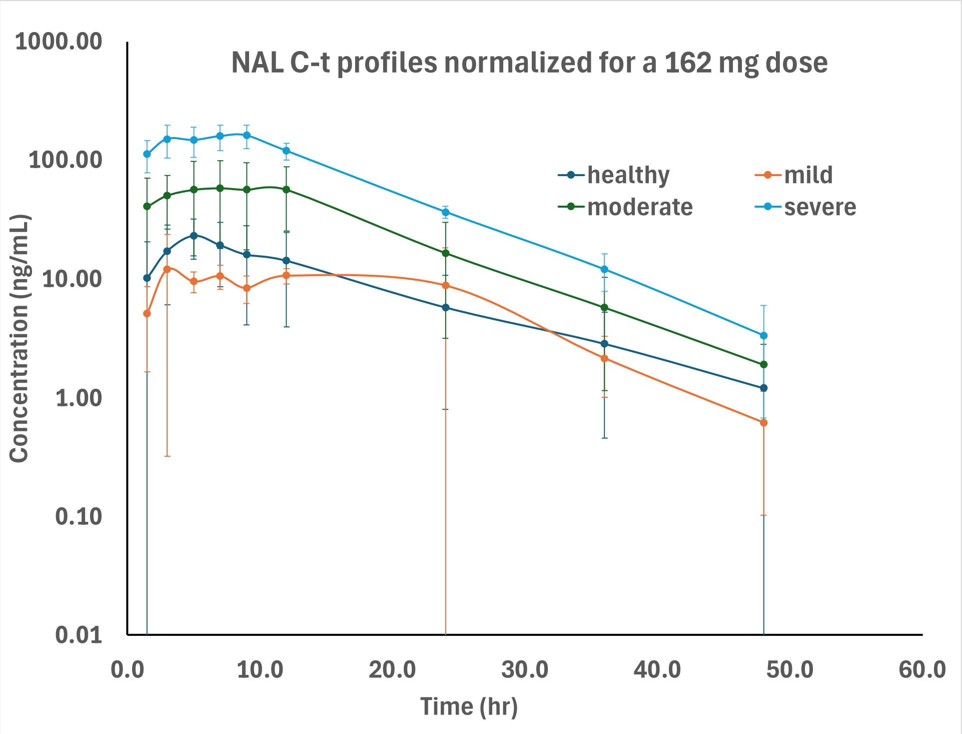

Figure S1: Concentration-time plots for nalbuphine in healthy and hepatic impairment groups. Average C-t NAL profiles (Mean ± SD) are reported for healthy subjects, and patients with mild, moderate, or severe hepatic impairment. Data are reported after normalization to a 162 mg dose.

II] Mathematica code for the PDE-EHR model, with moderate group datasets as example input.

See Code below.

In[1976]:=

```
ClearAll["Global`*"];
```

Drug specific parameters

In[1977]:=

```
{drug, f, fabs, fabscol, papp0, sol1, sol2, rho, difc, psize, fumics, doseoral,
   vol0, doseiv, inftime, xp2, ph0, pkaa, pkab, ma, mb, acidflag, baseflag} =
  {"Nalbuphine", 0.28, 100., 100., 16., 35., 35., 1.32, 2.6 * 10 ^ - 6, 50.,
   0.73, 163.3, 250., 18.1, 0.001, 4., 7.4, 14., 8.71, 4., 4., 0., 1.};
```

Determine drug PK for an IV dose.

In[1978]:=

```
ClearAll[modelpkiv1, modelpkiv2]
```

data in hr, ug/mL

In[1979]:=

```
inftime = 0.005;
```

In[1980]:=

```
ivpts = 15
```

Out[1980]=

```
15
```

In[1981]:=

```
dataIV = {{0.0166667, 354.896}, {0.0333333, 186.223}, {0.0833333, 116.995},
   {0.166667, 81.6422}, {0.25, 71.3518}, {0.5, 53.6919}, {0.75, 49.8407},
   {1., 45.581}, {1.5, 35.9013}, {2., 32.8557}, {4., 15.833},
   {6., 8.9992}, {8., 5.03928}, {10., 3.37814}, {12., 2.7521}};
```

In[1982]:=

```
ListLogPlot[dataIV]
```

Out[1982]=

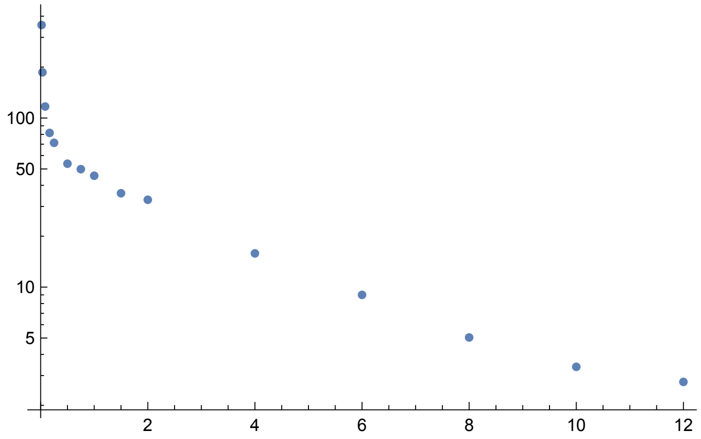

In[1983]:=

```
colort = {Red, Blue, Orange, Green, Purple, Cyan, Brown};
```

In[1984]:=

```
k12init = 1.1;
k21init = 2.5;
k13init = 9.5;
k31init = 2.7;
k10init = 2.3;
v1init = 51.5;
kinf = 1000 doseiv / inftime
```

Out[1990]=

3.62×10^{6}

In[1991]:=

```
ClearAll[k12, k21, k13, k31, k10, v1, modelpkiv1, fitiv];
modelpkiv1[k12_?NumericQ, k21_?NumericQ, k13_?NumericQ,
  k31_?NumericQ, k10_?NumericQ, v1_?NumericQ, te_?NumericQ] :=
 (model1[k12, k21, k13, k31, k10, v1][t] = (Xc[te] / v1) /.
   First[NDSolve[{Xc'[t] == k0[t] - (k12 + k13 + k10) Xc[t] + k21 Xp1[t] + k31 Xp2[t],
      Xp1'[t] == k12 Xc[t] - k21 Xp1[t],
      Xp2'[t] == k13 Xc[t] - k31 Xp2[t],
      k0'[t] == 0,
      Xc[0] == 0,
      Xp1[0] == 0,
      Xp2[0] == 0,
      k0[0] == kinf, WhenEvent[t == inftime, k0[t] → 0.]},
    {Xc, Xp1, Xp2}, {t, 0, 1.5 dataIV⟦ivpts, 1⟧},
     MaxSteps → 100000(*, PrecisionGoal → ∞*)]])
```

In[1993]:=

```
fitiv = NonlinearModelFit[dataIV,
  modelpkiv1[k12, k21, k13, k31, k10, v1, te], {{k12, k12init}, {k21, k21init},
   {k13, k13init}, {k31, k31init}, {k10, k10init}, {v1, v1init}},
  {te}, (*PrecisionGoal→∞,MaxIterations→10000,*)Weights → (1 / #2 &)]
```

Out[1993]=

FittedModel[modelpkiv1 [23.8, 2.57, ≪17≫, ≪19≫, ≪18≫, ≪18≫, te]]

In[1994]:=

```
fitiv["ParameterTable"]
```

Out[1994]=

	Estimate	Standard Error	t-Statistic	P-Value
k12	23.8152	4.15056	5.73781	0.000280575
k21	2.57455	0.213313	12.0694	7.33078×10^{-7}
k13	49.7676	7.74919	6.4223	0.000122037
k31	23.4376	3.16921	7.39541	0.0000412345
k10	4.19282	0.528678	7.93077	0.000023725
v1	19.8819	2.58357	7.69551	0.0000301388

In[1995]:=

```
fitiv["RSquared"]
```

Out[1995]=

```
0.998748
```

In[1996]:=

```
fitiv["AICc"]
```

Out[1996]=

```
90.0502
```

In[1997]:=

```
TableForm[fitiv["CorrelationMatrix"]]
```

Out[1997]//TableForm=

1.	0.424927	0.722508	0.761952	0.82219	-0.855194
0.424927	1.	-0.200063	0.465196	-0.0452289	0.0381501
0.722508	-0.200063	1.	0.494287	0.918281	-0.941021
0.761952	0.465196	0.494287	1.	0.471306	-0.496294
0.82219	-0.0452289	0.918281	0.471306	1.	-0.975447
-0.855194	0.0381501	-0.941021	-0.496294	-0.975447	1.

In[1998]:=

```
k12 = fitiv["BestFitParameters"]⟦1, 2⟧;
```

In[1999]:=

```
k21 = fitiv["BestFitParameters"]⟦2, 2⟧;
```

In[2000]:=

```
k13 = fitiv["BestFitParameters"]⟦3, 2⟧;
```

In[2001]:=

```
k31 = fitiv["BestFitParameters"]⟦4, 2⟧;
```

In[2002]:=

```
k10 = fitiv["BestFitParameters"]⟦5, 2⟧;
```

In[2003]:=

```
v1 = fitiv["BestFitParameters"]⟦6, 2⟧;
```

In[2004]:=

```
fitiv["FitResiduals"]
```

Out[2004]=

```
{0.237379, -0.476218, 1.50961, -2.9702, 2.26013, 0.455204, 1.76145, 1.19379,
 -2.15192, 0.218703, -1.82724, -0.556955, -0.131663, 0.580085, 1.23804}
```

In[2005]:=

```
fitiv["PredictedResponse"]
```

Out[2005]=

```
{354.659, 186.699, 115.485, 84.6124, 69.0917, 53.2367, 48.0792,
 44.3872, 38.0532, 32.637, 17.6602, 9.55616, 5.17094, 2.79805, 1.51406}
```

In[2006]:=
```
ClearAll[modelpkiv2];
modelpkiv2 =
  First[NDSolve[{Xc'[t] == k0[t] - (k12 + k13 + k10) Xc[t] + k21 Xp1[t] + k31 Xp2[t],
     Xp1'[t] == k12 Xc[t] - k21 Xp1[t],
     Xp2'[t] == k13 Xc[t] - k31 Xp2[t],
     k0'[t] == 0,
     Xc[0] == 0,
     Xp1[0] == 0,
     Xp2[0] == 0,
     k0[0] == kinf, WhenEvent[t == inftime, k0[t] → 0.]},
   {Xc, Xp1, Xp2}, {t, 0, 200}, MaxSteps → 100 000(*, PrecisionGoal → ∞*)]];
```

In[2008]:=
```
cl1 = k10 v1
```

Out[2008]=
```
83.3611
```

In[2009]:=
```
cl2 = k10 va
```

Out[2009]=
```
4.19282 va
```

In[2010]:=
```
beta = 1 / 2 ((k12 + k21 + k10) - ((k12 + k21 + k10) ^ 2 - 4 k21 k10) ^ 0.5)
```

Out[2010]=
```
0.357138
```

In[2011]:=
```
plot1 = Plot[(Xc[t] / v1) /. modelpkiv2, {t, 0, 1.1 dataIV⟦ivpts, 1⟧},
   PlotRange → {{0, 1.1 dataIV⟦ivpts, 1⟧}, {0, 1.1 dataIV⟦1, 2⟧}},
   Frame → True, FrameStyle → Directive[Black, 14, Thickness[0.003]],
   LabelStyle → (FontFamily → "Arial"), FrameLabel → {"time, h", "ug/L"}];
```

In[2012]:=
```
plot2 = ListPlot[dataIV];
```

In[2013]:=
```
Show[plot1, plot2]
```

Out[2013]=

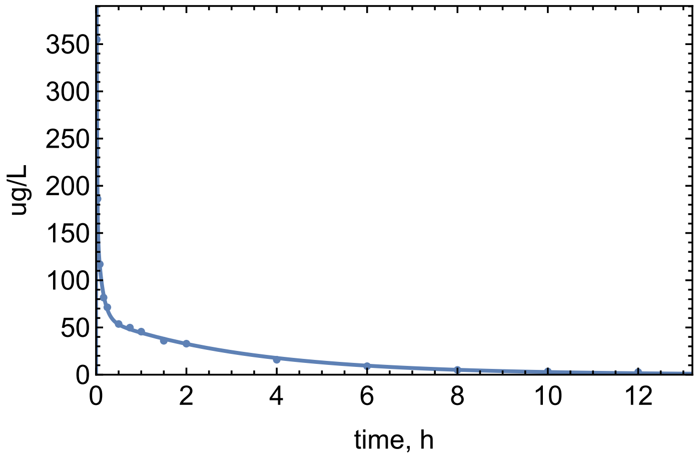

In[2014]:=

```
plot3 = LogPlot[(Xc[t] / v1) /. modelpkiv2, {t, 0, 1.1 dataIV⟦ivpts, 1⟧},
   PlotRange → {{0, 1.1 dataIV⟦ivpts, 1⟧}, {0.5 dataIV⟦ivpts, 2⟧, 2 dataIV⟦1, 2⟧}},
   Frame → True, FrameStyle → Directive[Black, 14, Thickness[0.003]],
   LabelStyle → (FontFamily → "Arial"), FrameLabel → {"time, h", "ug/L"}];
```

In[2015]:=

```
plot4 = ListLogPlot[dataIV];
```

In[2016]:=

```
Show[plot3, plot4]
```

Out[2016]=

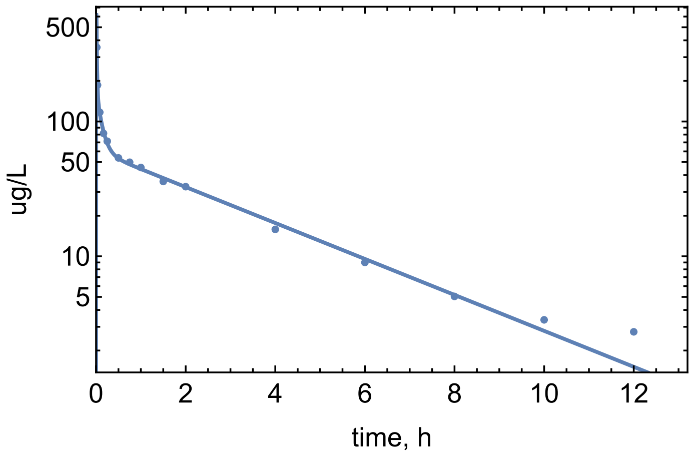

Oral dose modeling

Caco-2 scaling factor

In[2017]:=

```
cacosf = 1.1;
```

Fraction unbound in microsomes at 1 mg/mL

In[2018]:=

```
fumics
```

Out[2018]=

```
0.73
```

CACO2 Papp in m/hr (cm/sec*36 = m/hr)

In[2019]:=

```
papp0 = papp0 36 × 10 ^ - 6.
```

Out[2019]=

```
0.000576
```

In[2020]:=

```
papp0 = papp0 cacosf
```

Out[2020]=

```
0.0006336
```

Drug dose in mg for absorption model, ug for IV PK model, vol in m^3, infusion time in hr

In[2021]:=

```
vol0 = vol0 10 ^ - 6
```

Out[2021]=

```
0.00025
```

Concentration in Dosing solution (mg/m^3)

In[2022]:=

```
C0 = doseoral / vol0
```

Out[2022]=

```
653 200.
```

Intestinal radii (m)

In[2023]:=

```
r1 = 0.008;
r2 = 0.016;
```

Distances (m)

In[2025]:=

```
d0 = 0.05;
duo = 0.2;
jej = 1.5;
ile = 4.0;
```

---

cross sectional area in m^2

In[2029]:=

```
xa1 = π r1 ^ 2
```

Out[2029]=

```
0.000201062
```

In[2030]:=

```
xa2 = π r2 ^ 2
```

Out[2030]=

```
0.000804248
```

In[2031]:=

```
xa[x] = xa1 + (xa2 - xa1) / (1 + e^(-25 (x-ile)));
```

In[2032]:=

```
Plot[Evaluate[xa[x]], {x, 0, 10}, PlotRange → {{0, 10}, {0, 0.0022}}]
```

Out[2032]=

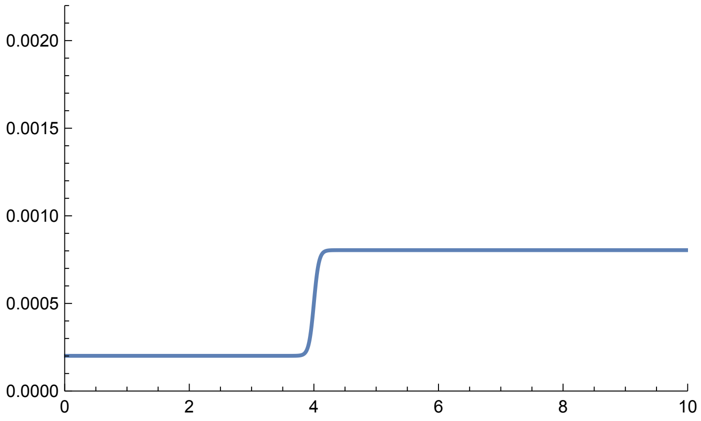

In[2033]:=

```
dxa[x] = D[xa[x], x];
```

In[2034]:=

```
sa1 = 3.9;
sa2 = 7.9;
sa3 = 4.5;
sa4 = 0.95;
```

In[2038]:=

```
sa[x] = sa1 + (sa2 - sa1) / (1 + e^(-50 (x-(d0+duo)/2))) -
    (sa2 - sa3) / (1 + e^(-50 (x-jej))) - (sa3 - sa4) / (1 + e^(-25 (x-ile)));
```

In[2039]:=

```
sa[x] /. x → 0
```

Out[2039]=

```
3.90771
```

In[2040]:=

```
Plot[Evaluate[sa[x]], {x, 0, 10}, PlotRange → {{0, 8}, {0, 16}}]
```

Out[2040]=

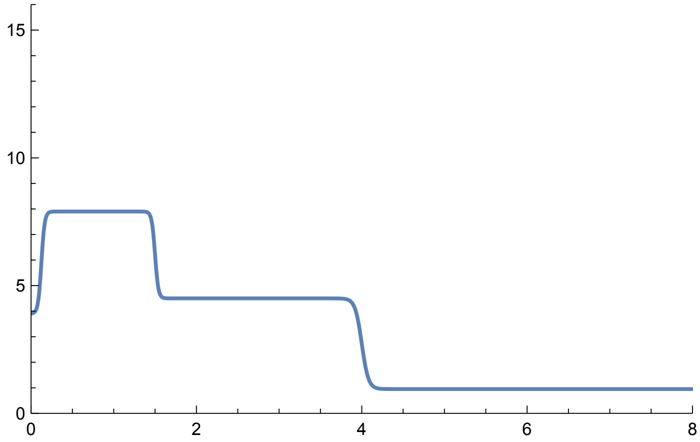

microvilli factor as a function of x

In[2041]:=

```
mf1 = 9.2;
mf2 = 14.1;
mf3 = 15.7;
mf4 = 6.6;
```

In[2045]:=

$$mf[x] = mf1 + (mf2 - mf1) \Big/ \left(1 + e^{-50\,(x-(d0+duo)/2)}\right) - (mf2 - mf3) \Big/ \left(1 + e^{-50\,(x-jej)}\right) - (mf3 - mf4) \Big/ \left(1 + e^{-25\,(x-ile)}\right);$$

In[2046]:=

```
mf[x] /. x → 0
```

Out[2046]=

```
9.20944
```

In[2047]:=

```
Plot[Evaluate[mf[x]], {x, 0, 10}, PlotRange → {{0, 8}, {0, 16}}]
```

Out[2047]=

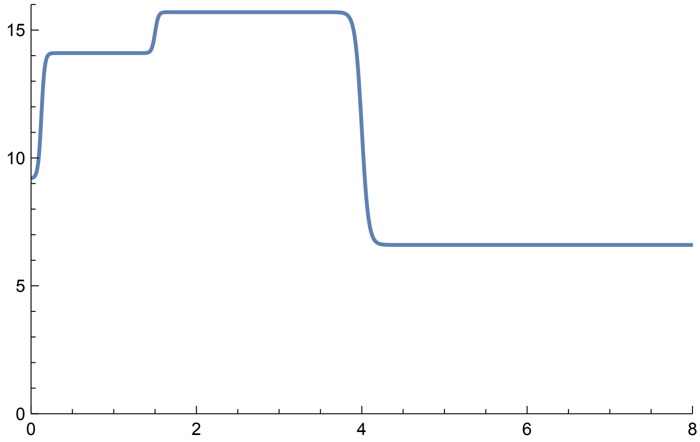

Cross sectional areas of enterocytes

In[2048]:=

```
xacell[x] = sa[x] / mf[x] 0.000020;
xamem[x] = sa[x] 0.0000000035;
xalip[x] = sa[x] / mf[x] 0.0000015;
```

Velocity function

In[2051]:=

```
ClearAll[x, velt, vplot1, vplot2];
```

In[2052]:=

```
velt = {{0, 5.7}, {0.2, 5.5}, {0.4, 5}, {0.7, 4},
   {1, 2.7}, {1.5, 1.5}, {2, 0.95}, {2.5, 0.8}, {3, 0.8}, {3.5, 0.8}};
```

In[2053]:=

```
vplot1 = ListPlot[velt, PlotRange → {{0, 12}, {0, 8}}];
```

In[2054]:=

```
vel[x] =
  0.5 (1 - Tanh[20. (x - 7.0)]) (6.0 - (6.0 - 0.5) / (1 + ⅇ^(-3 (x-1))) + (0.7) / (1 + ⅇ^(-6 (x-3.0))) -
     0.88 / (1 + ⅇ^(-25 (x-ile))) - (0.17) / (1 + ⅇ^(-3 (x-4.75))) - (0.15) / (1 + ⅇ^(-6 (x-6.8))));
```

In[2055]:=

```
vel0 = vel[x] /. x → 0
```

Out[2055]=

```
5.73916
```

In[2056]:=

```
vel[x] /. x → 8
```

Out[2056]=

```
0.
```

In[2057]:=

```
Plot[Evaluate[vel[x]], {x, 0, 8}]
```

Out[2057]=

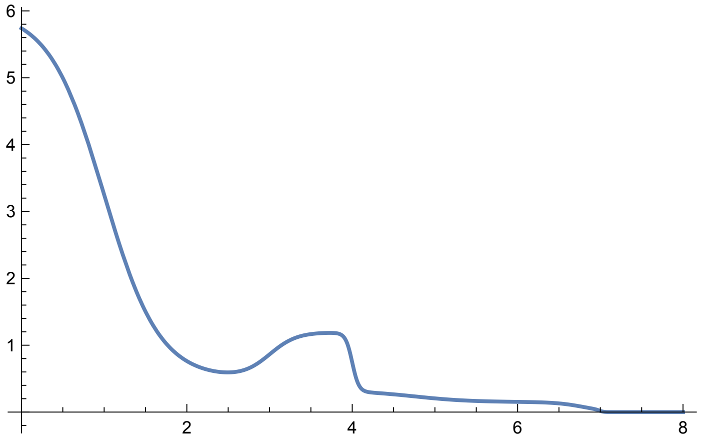

In[2058]:=

```
dvel[x] = D[vel[x], x];
```

Diffusion rate constant

effective diffusion in m^2/hr

In[2059]:=
```
slopegen = 3500;
```
In[2060]:=
```
diflag = 0.01;
```
In[2061]:=
```
dif2[x] = 0.02 vel[x] (LogisticSigmoid[slopegen (x - diflag)] -
    LogisticSigmoid[slopegen (x - (8. - diflag))]);
```
In[2062]:=
```
dif2[x] /. x → 0
```
Out[2062]=

7.23721×10^{-17}

In[2063]:=
```
ddif2[x] = D[dif2[x], x];
```

pH(x)

In[2064]:=
```
pH1 = 6.1;
```
In[2065]:=

$ph[x] = pH1 + 1.1 / \left(1 + e^{-2 (x-(2.0))}\right) - 0.8 / \left(1 + e^{-50 (x-4.)}\right) + 0.4 / \left(1 + e^{-5 (x-4.5)}\right);$

In[2066]:=
```
Plot[Evaluate[ph[x]], {x, 0, 10}, PlotRange → {{0, 8}, {5, 8}}]
```
Out[2066]=

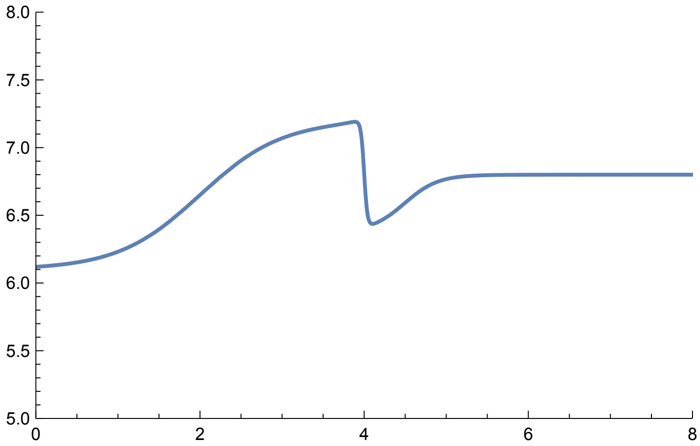

pH-permeability relationships

In[2067]:=
```
{ma, mb}
```
Out[2067]=
```
{4., 4.}
```

In[2068]:=

```
fn[x] =
 1 / (1 + acidflag (10 ^ (ma / 10 (ph[x] - pkaa))) + baseflag (10 ^ (mb / 10 (pkab - ph[x]))))
```

Out[2068]=

$$\frac{1}{1. + 1.\ 10^{0.4\left(2.61-\frac{0.4}{1+e^{-5(-4.5+x)}}+\frac{0.8}{1+e^{-50(-4.+x)}}-\frac{1.1}{1+e^{-2(-2.+x)}}\right)}}$$

In[2069]:=

```
fn0 = 1 / (1 + acidflag (10 ^ (ma / 10 (ph0 - pkaa))) + baseflag (10 ^ (mb / 10 (pkab - ph0))))
```

Out[2069]=

0.230311

In[2070]:=

```
p = papp0 / fn0
```

Out[2070]=

0.00275106

In[2071]:=

```
papp[x] = p fn[x]
```

Out[2071]=

$$\frac{0.00275106}{1. + 1.\ 10^{0.4\left(2.61-\frac{0.4}{1+e^{-5(-4.5+x)}}+\frac{0.8}{1+e^{-50(-4.+x)}}-\frac{1.1}{1+e^{-2(-2.+x)}}\right)}}$$

In[2072]:=

```
Plot[Evaluate[papp[x]], {x, 0, 10}]
```

Out[2072]=

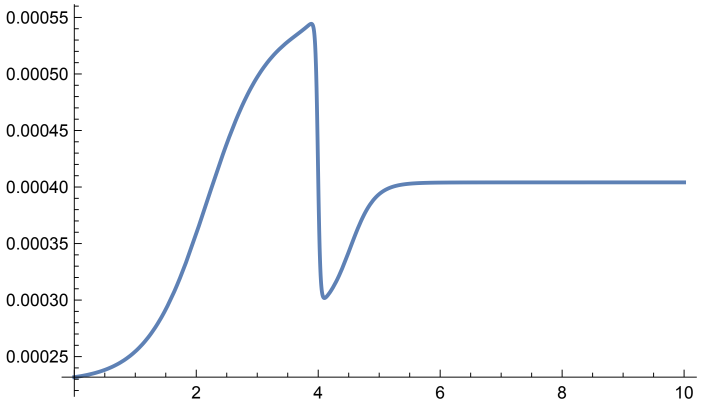

Slow permeability due to decreased radial diffusion

In[2073]:=

```
ClearAll[difr1]
```

In[2074]:=

```
difr1[x] = 0.5 (1 - Tanh[4. (x - 6.5)]);
```

In[2075]:=

```
Plot[Evaluate[difr1[x]], {x, 0, 10}, PlotRange → {{0, 8}, {0, 1.1}}]
```

Out[2075]=

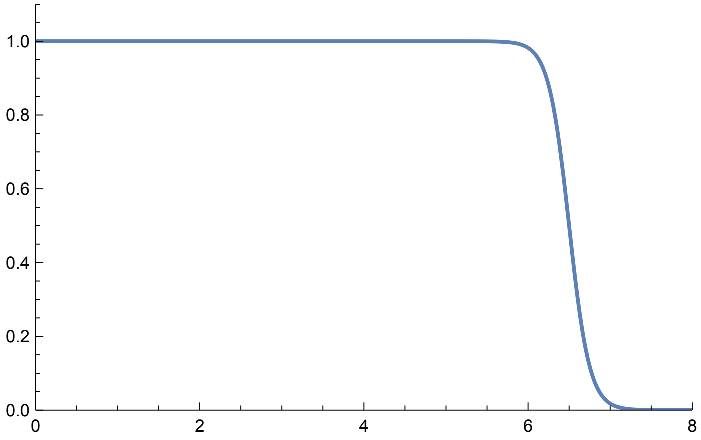

In[2076]:=

```
papp2[x] = difr1[x] × papp[x];
```

In[2077]:=

```
Plot[Evaluate[papp2[x]], {x, 0, 10}, PlotRange → {{0, 8}, {0, 0.000025}}]
```

Out[2077]=

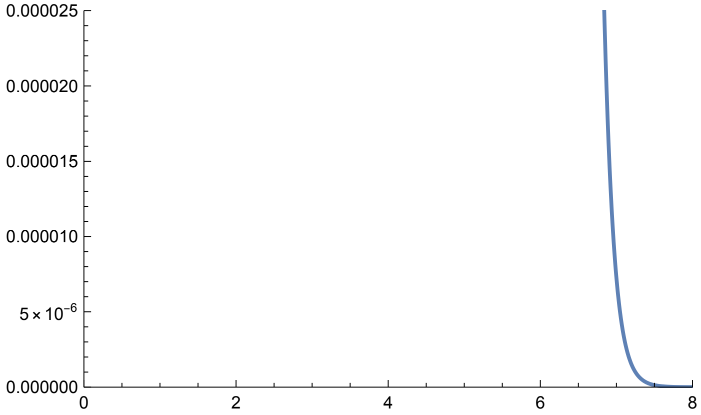

Drug Parameters

Partition coefficient based on 0.7 uL lipid per 1 mg microsomal protein

In[2078]:=

```
Kp = ((1 - fumics) / fumics) (1 / 0.0007)
```

Out[2078]=

```
528.376
```

In[2079]:=

```
cli[x] =
  (UnitStep[ (x - (diflag))] - UnitStep[x - (lintest - (diflag))]) 4 papp[x] × sa[x];
```

In[2080]:=

```
clo[x] = (UnitStep[ (x - (diflag))] - UnitStep[x - (lintest - (diflag))]) cli[x] / Kp;
```

In[2081]:=

```
cli2[x] =
  (UnitStep[ (x - (diflag))] - UnitStep[x - (lintest - (diflag))]) 4 papp2[x] × sa[x];
```

In[2082]:=
```
clo2[x] = (UnitStep[ (x - (diflag))] -  UnitStep[x - (lintest - (diflag))]) cli2[x] / Kp;
```
In[2083]:=
```
cli3[x] =
  (UnitStep[ (x - (diflag))] -  UnitStep[x - (lintest - (diflag))]) 4 papp[x] 100 sa[x];
clo3[x] = (UnitStep[ (x - (diflag))] -  UnitStep[x - (lintest - (diflag))]) cli3[x] / Kp;
```

Stomach pulse length time in m /(m/hr), and lag time (hr)

In[2085]:=
```
pl1 = 0.3;
```
In[2086]:=
```
lag = 0.1;
```

In[2087]:=
```
volcor = vol0 / (π r1^2 pl1 vel0)
```
Out[2087]=
```
0.722172
```

Use a smoothed pulse input function

In[2088]:=
```
upulse1[t] = (LogisticSigmoid[180 (t - lag)] - LogisticSigmoid[180 (t - (pl1 + lag))])
```
Out[2088]=
```
-LogisticSigmoid[180 (-0.4 + t)] + LogisticSigmoid[180 (-0.1 + t)]
```
In[2089]:=
```
Plot[Evaluate[upulse1[t]], {t, 0, 10}, PlotRange → {{0, 2}, {0, 1.1}}]
```
Out[2089]=

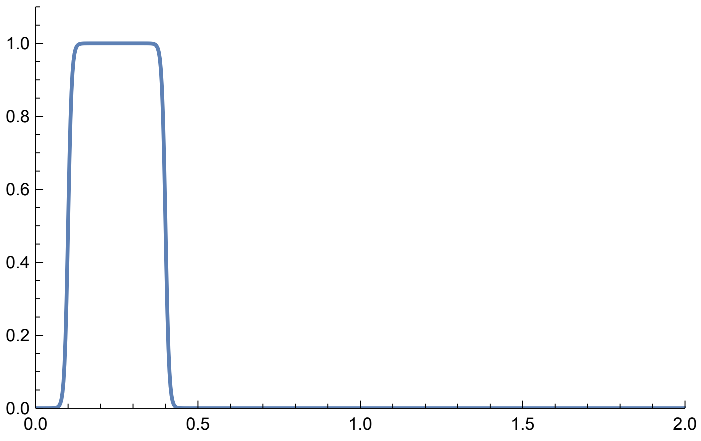

In[2090]:=
```
pulse1[t] = C0 volcor upulse1[t];
```
In[2091]:=
```
Plot[Evaluate[pulse1[t]], {t, 0, 10}, PlotRange → {{0, 2}, {0, 1.1 doseoral / vol0}}]
```

Out[2091]=

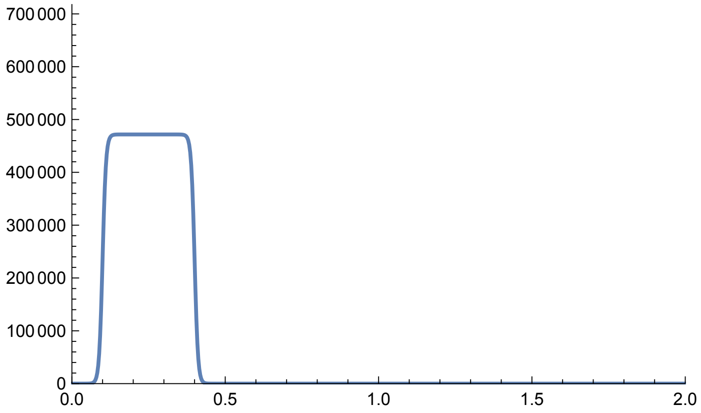

In[2092]:=

```
NIntegrate[pulse1[t] vel0 xa1, {t, 0, 1.0}]
```

Out[2092]=

```
163.3
```

In[2093]:=

```
c10 = Evaluate[pulse1[t] /. t → 0]
```

Out[2093]=

```
0.00718433
```

Dissolution in the stomach; first order dissolution is assumed

In[2094]:=

```
Clear[models1, tmax]
```

In[2095]:=

```
kdiss = 0.13;
```

In[2096]:=

```
models1 = NDSolve[{
      vol'[t] == -(upulse1[t] / pl1) vol0,
      Css'[t] == -UnitStep[t - 0.01] kdiss Css[t],
      Cds'[t] == UnitStep[t - 0.01] kdiss Css[t],
      vol[0] == vol0,
      Css[0] == doseoral / vol0,
      Cds[0] == 0},
     {vol, Css, Cds}, {t, 0, 25}, MaxSteps → 1 000 000, PrecisionGoal → 13][[1]];
```

In[2097]:=

```
Plot[vol[t] /. models1, {t, 0, 1}]
```

Out[2097]=

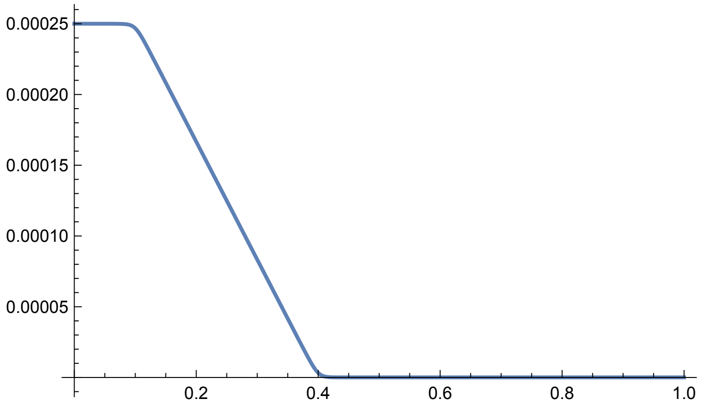

In[2098]:=

```
Plot[Css[t] × vol[t] /. models1, {t, 0, 1}]
```

Out[2098]=

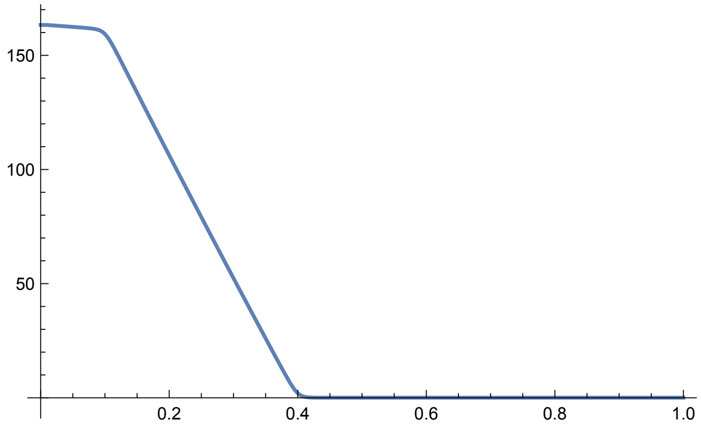

In[2099]:=

```
pulsess1[t] = upulse1[t] volcor Css[t] /. models1;
```

In[2100]:=

```
Plot[Evaluate[pulsess1[t]], {t, 0, 1}]
```

Out[2100]=

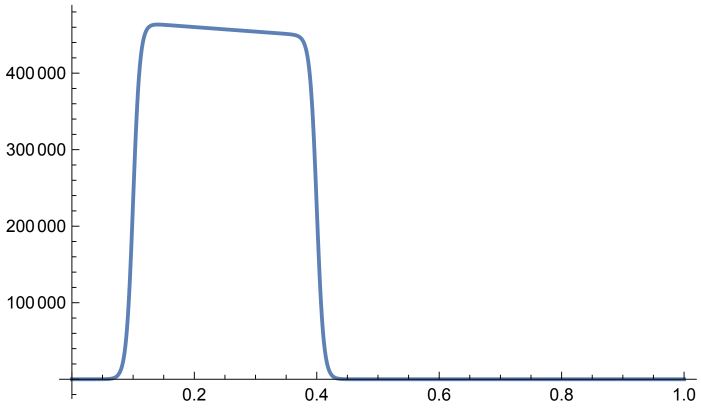

In[2101]:=

```
cs0 = Evaluate[(pulsess1[t]) /. t → 0]
```

Out[2101]=

```
0.00718433
```

In[2102]:=

```
intpulsess1 = NIntegrate[pulsess1[t] vel0 xa1, {t, 0, 20.0}]
```

Out[2102]=

```
158.294
```

In[2103]:=

```
xa1
```

Out[2103]=

```
0.000201062
```

In[2104]:=

```
pulseds1[t] = upulse1[t] volcor Cds[t] /. models1;
```

In[2105]:=

```
Plot[Evaluate[pulseds1[t]], {t, 0, 1}]
```

Out[2105]=

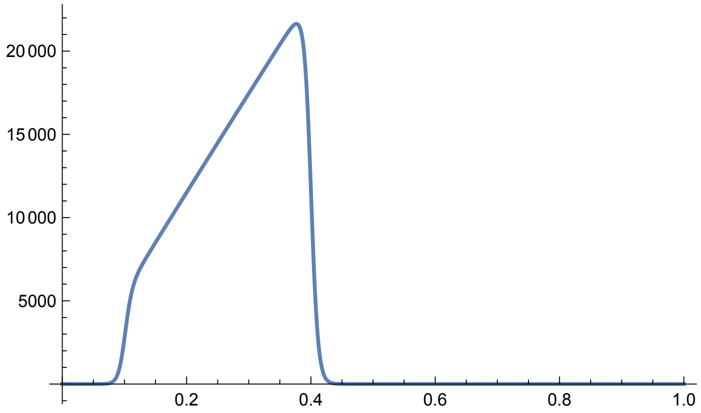

In[2106]:=

```
c0 = Evaluate[pulseds1[t] /. t → 0]
```

Out[2106]=

```
0.
```

In[2107]:=

```
intpulseds1 = NIntegrate[pulseds1[t] vel0 xa1, {t, 0, 20.0}]
```

Out[2107]=

```
5.00613
```

Total inputs should equal dose

In[2108]:=

```
intpulsess1 + intpulseds1
```

Out[2108]=

```
163.3
```

Convert PK ODEs to PDEs

C in ug/L = mg/m^3

In[2109]:=
```
va = v1 / 1000;
```
In[2110]:=
```
lintest = 8.0;
```
In[2111]:=
```
xaa = va / lintest;
```
In[2112]:=
```
vb = k12 va / k21
```
Out[2112]=
```
0.183912
```
In[2113]:=
```
xab = vb / lintest;
```
In[2114]:=
```
cld2 = k12 va
```
Out[2114]=
```
0.47349
```
In[2115]:=
```
vc = k13 va / k31;
```
In[2116]:=
```
xac = vc / lintest;
```
In[2117]:=
```
cld3 = k13 va;
```
In[2118]:=
```
cl1 = k10 va
```
Out[2118]=
```
0.0833611
```

Use ecf = 15 for m5 central comp

In[2119]:=
```
vm5a = 15. / 1000;
```
In[2120]:=
```
xam5a = vm5a / lintest;
```

Use 0.8 1C for m3, vam3=va+vb+vc

In[2121]:=
```
vm3a = 0.8 (va + vb + vc);
```
In[2122]:=
```
xam3a = vm3a / lintest;
```

Use 0.8 1C for m4, vam4=va+vb+vc

In[2123]:=
```
vm4a = 0.8 (va + vb + vc);
```
In[2124]:=
```
xam4a = vm4a / lintest;
```

Use 0.2 1C for m1, vam3=va+vb+vc

In[2125]:=
```
vm1a = 0.2 (va + vb + vc);
```

In[2126]:=
```
xam1a = vm1a / lintest;
```

gall bladder - 16mL

In[2127]:=
```
vgb = 0.000016;
xagb = vgb / lintest;
```

gall bladder - 16mL

In[2129]:=
```
vgbm4g = 0.000016;
xagbm4g = vgbm4g / lintest;
```

Liver

In[2131]:=
```
bp = 1.15;
```

In[2132]:=
```
bpm5 = 0.56;
```

In[2133]:=
```
bpm3 = 0.9;
bpm1 = 0.7;
```

In[2135]:=
```
bpm4 = 0.9;
```

In[2136]:=
```
qliv1 = 1.451 × 60 / 1000
```

Out[2136]=
```
0.08706
```

In[2137]:=
```
vliv = 0.00182;
xaliv = vliv / lintest;
```

In[2139]:=
```
vlivm3 = 0.00182;
xalivm3 = vlivm3 / lintest;
```

In[2141]:=
```
vlivm4 = 0.00182;
xalivm4 = vlivm4 / lintest;
```

In[2143]:=
```
ClearAll[clh]
```

Clearance definitions
CLnh = nonhepatic clearance

CLh = hepatic clearance

CLint = intrinsic hepatic clearance

In[2144]:=
```
clnh1 = 0.00414;
```

In[2145]:=
```
fg = 1.0;
```

In[2146]:=
```
er = 1 - f / fg
```

Out[2146]=
```
0.72
```

In[2147]:=
```
solnclh = Solve[er == clh / (bp qliv1), clh][[1]]
```

Out[2147]=
```
{clh → 0.0720857}
```

In[2148]:=
```
clh1 = clh /. solnclh
```

Out[2148]=
```
0.0720857
```

In[2149]:=
```
clh1 = clh1 / 1.4
```

Out[2149]=
```
0.0514898
```

In[2150]:=
```
soln = Solve[clh1 / bp == qliv1 clint / (qliv1 + clint), clint][[1]]
```

Out[2150]=
```
{clint → 0.0921812}
```

In[2151]:=
```
clint1 = clint /. soln
```

Out[2151]=
```
0.0921812
```

Formation clearances of metabolites

In[2152]:=
```
clintm31 = 0.04;
```

In[2153]:=
```
clintm4g1 = 0.005;
```

Elimination clearances of metabolites

In[2154]:=
```
clm51 = 0.017;
```

In[2155]:=
```
clm31 = 0.004;
clm11 = 0.02;
clm41 = 0.04;
```

In[2158]:=

```
clinth[x] = clint1 (UnitStep[ (x - diflag)] - UnitStep[x - (lintest - diflag)]);
```

In[2159]:=

```
Plot[Evaluate[clinth[x]], {x, 0, lintest},
 PlotRange → {{0, lintest}, {0, 1.1 clint1}}]
```

Out[2159]=

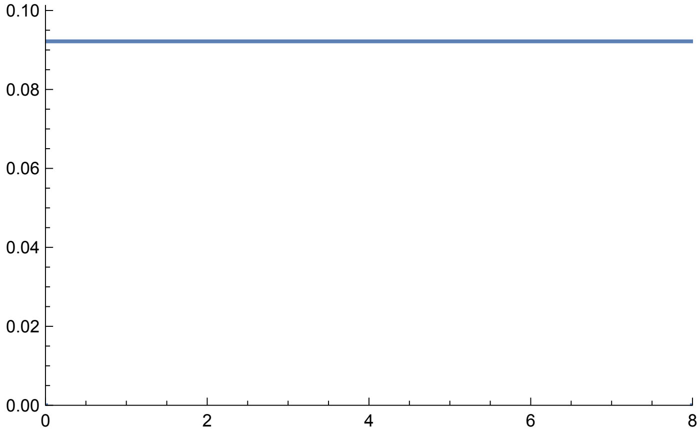

In[2160]:=

```
clintm3[x] = clintm31 (UnitStep[ (x - diflag)] - UnitStep[x - (lintest - diflag)]);
```

In[2161]:=

```
Plot[Evaluate[clintm3[x]], {x, 0, lintest},
 PlotRange → {{0, lintest}, {0, 1.1 clintm3}}]
```

Out[2161]=

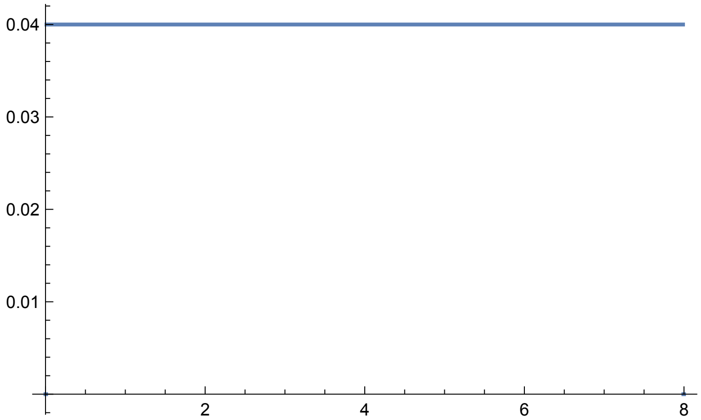

In[2162]:=

```
clintm4g[x] = clintm4g1 (UnitStep[ (x - diflag)] - UnitStep[x - (lintest - diflag)]);
```

In[2163]:=

```
Plot[Evaluate[clintm4g[x]], {x, 0, lintest},
 PlotRange → {{0, lintest}, {0, 1.1 clintm4g1}}]
```

Out[2163]=

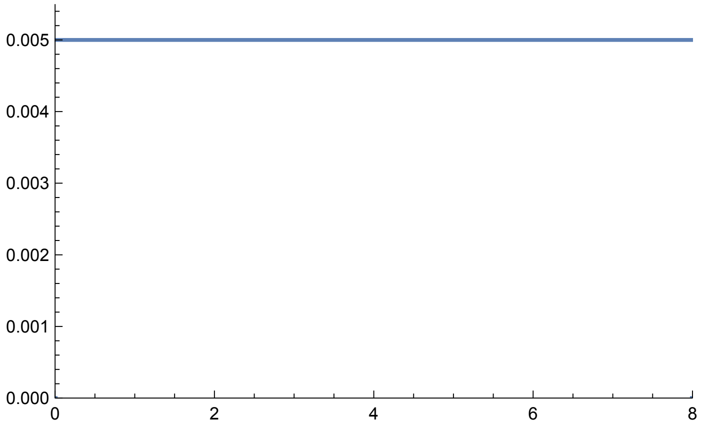

In[2164]:=

```
clnh[x] = clnh1 (UnitStep[ (x - diflag)] - UnitStep[x - (lintest - diflag)]);
```

In[2165]:=

```
Plot[Evaluate[clnh[x]], {x, 0, lintest}, PlotRange → {{0, lintest}, {0, 1.1 clnh1}}]
```

Out[2165]=

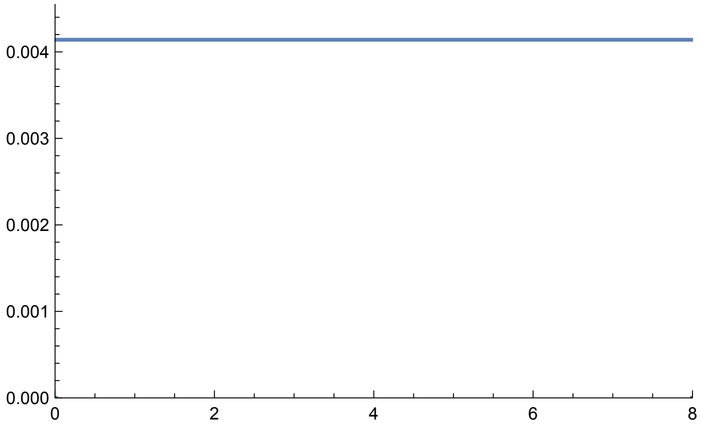

In[2166]:=

```
clm5[x] = clm51 (UnitStep[ (x - diflag)] - UnitStep[x - (lintest - diflag)]);
```

In[2167]:=

```
Plot[Evaluate[clm5[x]], {x, 0, lintest}, PlotRange → {{0, lintest}, {0, 1.1 clm51}}]
```

Out[2167]=

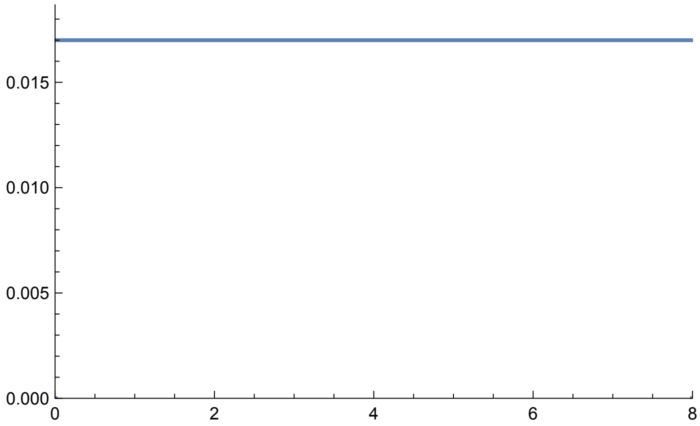

In[2168]:=

```
clm3[x] = clm31 (UnitStep[ (x - diflag)] - UnitStep[x - (lintest - diflag)]);
```

In[2169]:=

```
Plot[Evaluate[clm3[x]], {x, 0, lintest}, PlotRange → {{0, lintest}, {0, 1.1 clm31}}]
```

Out[2169]=

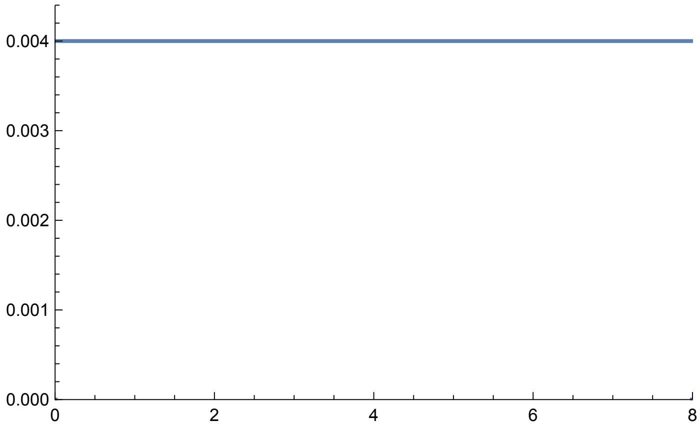

In[2170]:=

```
clm1[x] = clm11 (UnitStep[ (x - diflag)] - UnitStep[x - (lintest - diflag)]);
```

In[2171]:=

```
Plot[Evaluate[clm1[x]], {x, 0, lintest}, PlotRange → {{0, lintest}, {0, 1.1 clm11}}]
```

Out[2171]=

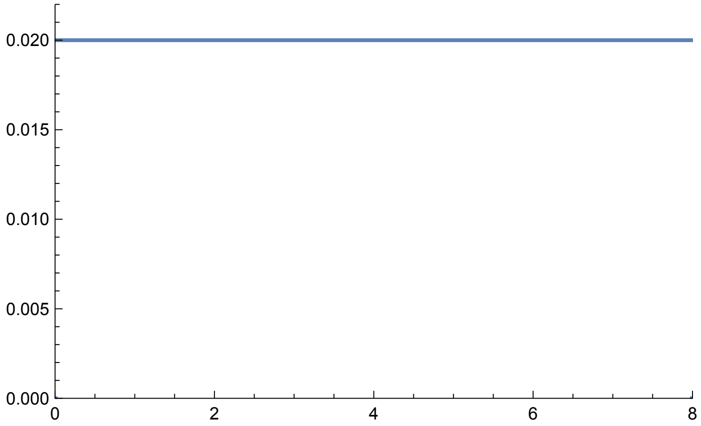

In[2172]:=

```
clm4[x] = clm41 (UnitStep[ (x - diflag)] - UnitStep[x - (lintest - diflag)]);
```

In[2173]:=

```
Plot[Evaluate[clm4[x]], {x, 0, lintest}, PlotRange → {{0, lintest}, {0, 1.1 clm41}}]
```

Out[2173]=

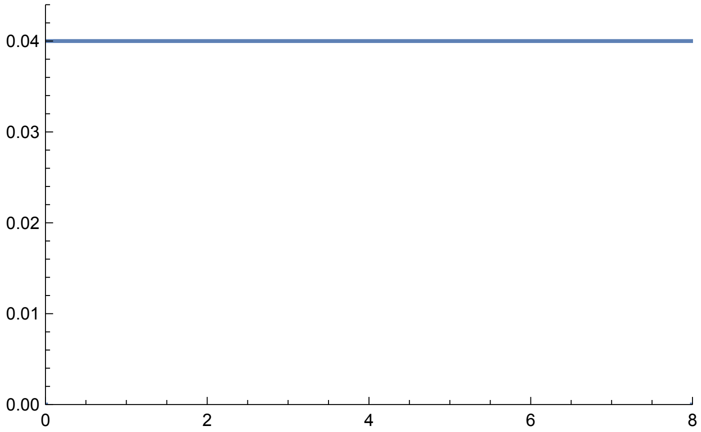

In[2174]:=

```
cldb[x] = cld2 (UnitStep[ (x - (diflag))] -  UnitStep[x - (lintest - (diflag))]);
```

In[2175]:=

```
Plot[Evaluate[cldb[x]], {x, 0, lintest}, PlotRange → {{0, lintest}, {0, 1.1 cld2}}]
```

Out[2175]=

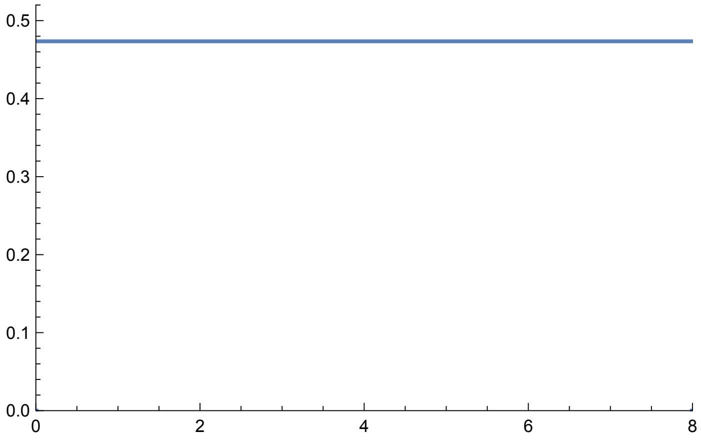

In[2176]:=

```
cldc[x] = cld3 (UnitStep[ (x - (diflag))] - UnitStep[x - (lintest - (diflag))]);
```

In[2177]:=

```
Plot[Evaluate[cldc[x]], {x, 0, lintest}, PlotRange → {{0, lintest}, {0, 1.1 cld3}}]
```

Out[2177]=

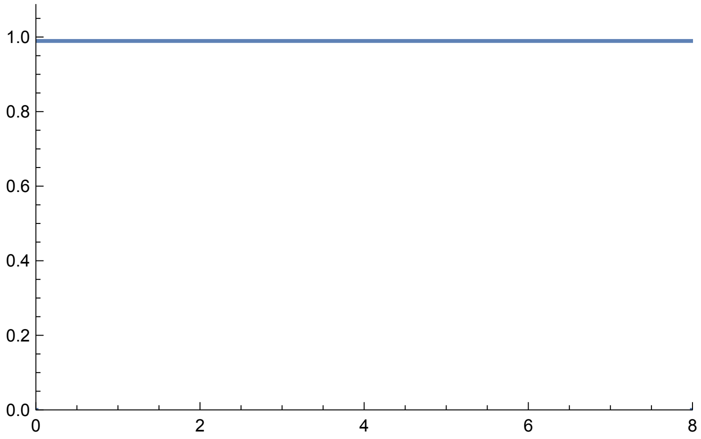

In[2178]:=

```
qliv[x] = qliv1 (UnitStep[ (x - (diflag))] - UnitStep[x - (lintest - (diflag))]);
```

In[2179]:=

```
Plot[Evaluate[qliv[x]], {x, 0, lintest}, PlotRange → {{0, lintest}, {0, 1.1 qliv1}}]
```

Out[2179]=

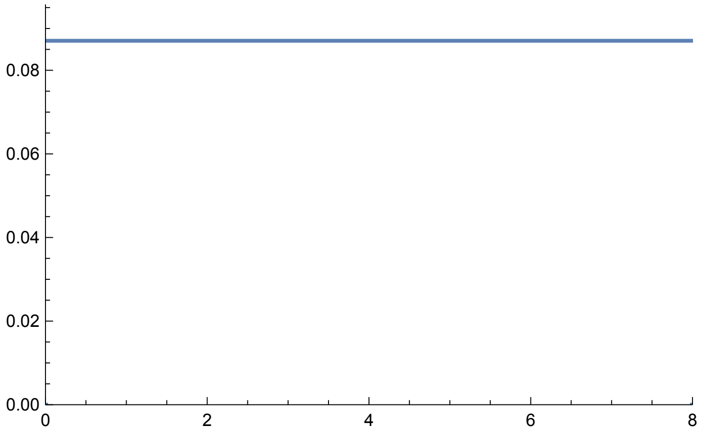

Parent + glucuronides into gall bladder from liver

In[2180]:=

```
fclgbp = 0.25;
```

In[2181]:=

```
fclgbm = 0.25;
```

In[2182]:=

```
clgip1 = clint1 fclgbp
```

Out[2182]=

```
0.0230453
```

In[2183]:=

```
clgim1 = clint1 fclgbm
```

Out[2183]=

```
0.0230453
```

In[2184]:=

```
clgip[x] = clgip1 (LogisticSigmoid[ slopegen (x - diflag)] -
      LogisticSigmoid[ slopegen (x - (lintest - diflag))]);
```

In[2185]:=

```
Plot[Evaluate[clgip[x]], {x, 0, lintest}, PlotRange → {{0, lintest}, {0, 1.1 clgip1}}]
```

Out[2185]=

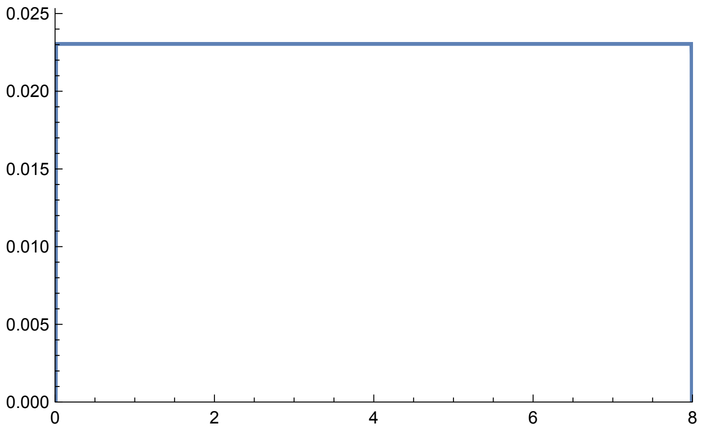

In[2186]:=

```
clgim[x] = clgim1 (LogisticSigmoid[ slopegen (x - diflag)] -
      LogisticSigmoid[ slopegen (x - (lintest - diflag))]);
```

In[2187]:=

```
Plot[Evaluate[clgim[x]], {x, 0, lintest}, PlotRange → {{0, lintest}, {0, 1.1 clgim1}}]
```

Out[2187]=

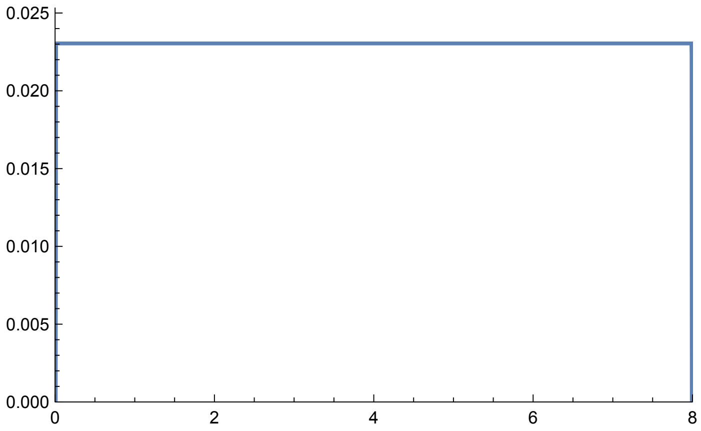

Where the bile enters the intestine

In[2188]:=

```
duomid = 0.1;
```

In[2189]:=

```
bdwidth = 0.01;
```

In[2190]:=

```
clgo1 = 0.0000065 clgip1
```

Out[2190]=

1.49794×10^{-7}

In[2191]:=

```
clgo[x] = (LogisticSigmoid[ slopegen (x - duomid)] -
     LogisticSigmoid[ slopegen (x - (duomid + bdwidth))]);
```

In[2192]:=

```
Plot[Evaluate[clgo[x]], {x, 0, 0.2}, PlotRange → {{0, 0.2}, {0, 1.1 }}]
```

Out[2192]=

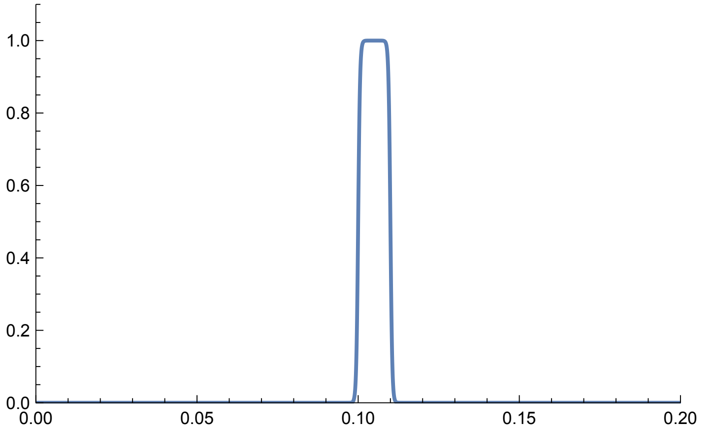

Feeding time function

In[2193]:=

```
ft1 = 3.;
```

In[2194]:=

```
ft2 = 9.;
```

In[2195]:=

```
ft3 = 20.;
```

In[2196]:=

```
pl1 = 40. / 60.;
```

In[2197]:=

```
clgo2[t] = 4.0 (vgb) (LogisticSigmoid[ 0.01 slopegen (t - ft1)] - LogisticSigmoid[
      0.01 slopegen (t - (ft1 + pl1))] + LogisticSigmoid[ 0.01 slopegen (t - ft2)] -
     LogisticSigmoid[ 0.01 slopegen (t - (ft2 + pl1))] + LogisticSigmoid[
      0.01 slopegen (t - ft3)] - LogisticSigmoid[ 0.01 slopegen (t - (ft3 + pl1))]);
```

In[2198]:=

```
Plot[Evaluate[clgo2[t]], {t, 2.5, 21.5},
 PlotRange → {{2.5, 21.5}, {-.00001, 4.1 (vgb)}}]
```

Out[2198]=

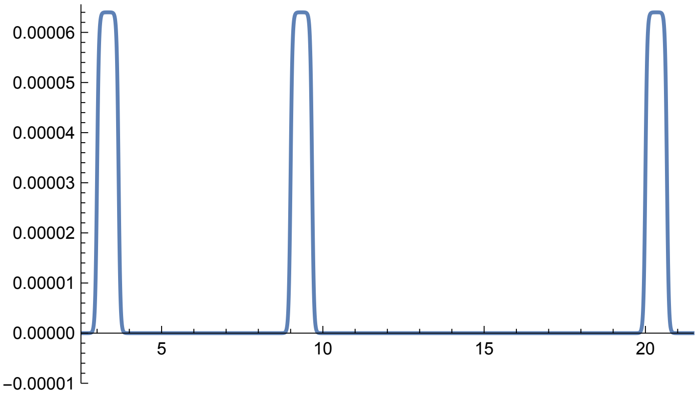

Glucuronidase function

In[2199]:=

```
clgus1 = 0.01
```

Out[2199]=

```
0.01
```

In[2200]:=

```
clgus[x] = clgus1 LogisticSigmoid[2. (x - 6.6)];
```

In[2201]:=

```
Plot[Evaluate[clgus[x]], {x, 0, 8}, PlotRange → {{0, 8}, {-.00001, 1.1 clgus1}}]
```

Out[2201]=

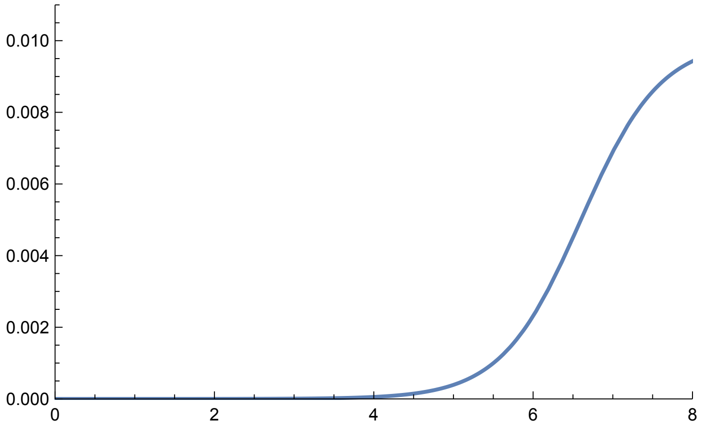

Artificial (mathematical) velocity

In[2202]:=

```
vel1[x] = (40. - 40. LogisticSigmoid[slopegen (x - diflag)] -
    40. LogisticSigmoid[slopegen (x - (lintest - diflag))]);
```

In[2203]:=

```
dvel1[x] = D[vel1[x], x];
```

In[2204]:=

```
Plot[Evaluate[vel1[x]], {x, 0, lintest}, PlotRange → {{0, lintest}, {-0.05 , 0.05}}]
```

Out[2204]=

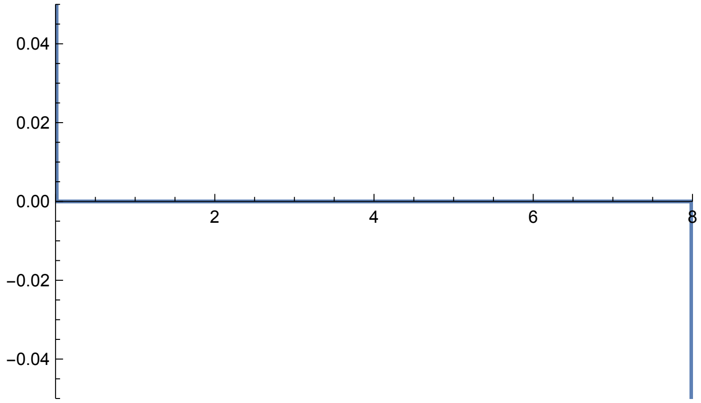

In[2205]:=
```
vel1[x] /. x → {0.001, 2., 7.999}
```
Out[2205]=
```
{40., 0., -40.}
```

Very rapid diffusion for non-intestinal compartments

In[2206]:=
```
dif1 = 700.;
```
In[2207]:=
```
dif1b[x] = dif1 (LogisticSigmoid[ slopegen (x - diflag)] -
      LogisticSigmoid[ slopegen (x - (lintest - diflag))]);
```
In[2208]:=
```
Plot[Evaluate[dif1b[x]], {x, 0, lintest}, PlotRange → {{0, lintest}, {0, 1.1 dif1}}]
```
Out[2208]=

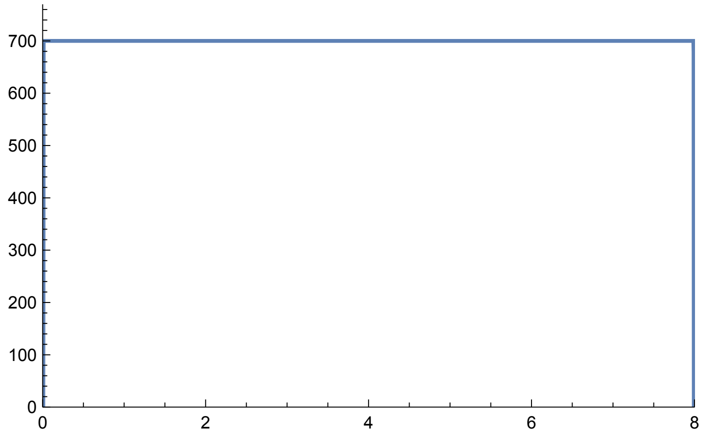

In[2209]:=
```
ddif1b[x] = D[dif1b[x], x];
```
In[2210]:=
```
dif1b[x] /. x → 7.999
```
Out[2210]=

1.46105×10^{-11}

Create a grid for Method of Lines

In[2211]:=
```
coordx = Join[0. + Range[0, 1500] / 15 000, 0.1 + Range[1, 560] / 400,
   1.5 + Range[1, 250] / 100, 4.0 + Range[1, 40] / 20, 6.0 + Range[1, 150] / 100,
   7.5 + Range[1, 4] / 10, 7.9 + Range[1, 1500] / 15 000];
```
In[2212]:=
```
Length[coordx]
```
Out[2212]=
```
4005
```

```
In[2213]:=
Max[coordx]
Out[2213]=
8.
```

Intestinal dissolution rate constant

```
In[2214]:=
kdis = 0.15;
```

Molecular weights

```
In[2215]:=
mwnal = 354.;
mwm5 = 354. + 176.;
In[2217]:=
mwm3 = 354. + 16.;
In[2218]:=
mwm1 = 354. + 48.;
In[2219]:=
mwm4 = 354. + 16.;
In[2220]:=
mwm4g = 354. + 16. + 176.;
```

PDE Model

```
In[2221]:=
soln = NDSolve[{
   D[Cs1[t, x], t] == dif2[x] × D[Cs1[t, x], {x, 2}] +
     (-vel[x] + ddif2[x] + dif2[x] × dxa[x] / xa[x]) D[Cs1[t, x], x] +
     Cs1[t, x] (-dvel[x] - (dxa[x] / xa[x]) vel[x]) - kdis Cs1[t, x],
   D[C1[t, x], t] == dif2[x] × D[C1[t, x], {x, 2}] +
     (-vel[x] + ddif2[x] + dif2[x] × dxa[x] / xa[x]) D[C1[t, x], x] + C1[t, x]
      (-dvel[x] - (dxa[x] / xa[x]) vel[x]) + kdis Cs1[t, x] - cli2[x] × C1[t, x] / xa[x] +
     clo2[x] × C2[t, x] / xa[x] + clgo1 clgo[x] / bdwidth × Cgb[t, x] / xa[x] +
     clgo2[t] × clgo[x] / bdwidth × Cgb[t, x] / xa[x] + clgus[x] × C1m5[t, x] / xa[x],
   D[C1m5[t, x], t] == dif2[x] × D[C1m5[t, x], {x, 2}] +
     (-vel[x] + ddif2[x] + dif2[x] × dxa[x] / xa[x]) D[C1m5[t, x], x] +
     C1m5[t, x] (-dvel[x] - (dxa[x] / xa[x]) vel[x]) +
     clgo1 clgo[x] / bdwidth × Cgbm[t, x] / xa[x] +
     clgo2[t] × clgo[x] / bdwidth × Cgbm[t, x] / xa[x] - clgus[x] × C1m5[t, x] / xa[x],
   D[C1m4g[t, x], t] == dif2[x] × D[C1m4g[t, x], {x, 2}] +
     (-vel[x] + ddif2[x] + dif2[x] × dxa[x] / xa[x]) D[C1m4g[t, x], x] +
     C1m4g[t, x] (-dvel[x] - (dxa[x] / xa[x]) vel[x]) +
     clgo1 clgo[x] / bdwidth × Cgbm4g[t, x] / xa[x] +
     clgo2[t] × clgo[x] / bdwidth × Cgbm4g[t, x] / xa[x] - clgus[x] × C1m4g[t, x] / xa[x],
   D[C2[t, x], t] == cli2[x] × C1[t, x] / xamem[x] -
```

```
  (clo[x] + clo2[x]) C2[t, x] / xamem[x] + cli[x] × C3[t, x] / xamem[x],
D[C3[t, x], t] == clo[x] × C2[t, x] / xacell[x] - cli[x] × C3[t, x] / xacell[x] -
  cli3[x] × C3[t, x] / xacell[x] + clo3[x] × C4[t, x] / xacell[x] -
  (cli[x] / 2) C3[t, x] / xacell[x],
D[C4[t, x], t] == -clo3[x] × C4[t, x] / xalip[x] + cli3[x] × C3[t, x] / xalip[x],
D[Ca[t, x], t] ==
 dif1b[x] × D[Ca[t, x], {x, 2}] + (ddif1b[x] - vel1[x]) D[Ca[t, x], x] -
  dvel1[x] × Ca[t, x] - cldb[x] / (lintest - 2 diflag) × Ca[t, x] / xaa +
  cldb[x] / (lintest - 2 diflag) × Cb[t, x] / xaa - cldc[x] / (lintest - 2 diflag) ×
   Ca[t, x] / xaa + cldc[x] / (lintest - 2 diflag) × Cc[t, x] / xaa -
  qliv[x] / (lintest - 2 diflag) bp Ca[t, x] / xaa + qliv[x] / (lintest - 2 diflag) ×
   Cliv[t, x] / xaa - clnh[x] / (lintest - 2 diflag) bp Ca[t, x] / xaa,
D[Cam5[t, x], t] ==
 dif1b[x] × D[Cam5[t, x], {x, 2}] + (ddif1b[x] - vel1[x]) D[Cam5[t, x], x] -
  dvel1[x] × Cam5[t, x] - clm5[x] / (lintest - 2 diflag) bpm5 Cam5[t, x] / xam5a +
  (mwm5 / mwnal) (0.55) clinth[x] / (lintest - 2 diflag) × Cliv[t, x] / xam5a,
D[Cam3[t, x], t] ==
 dif1b[x] × D[Cam3[t, x], {x, 2}] + (ddif1b[x] - vel1[x]) D[Cam3[t, x], x] -
  dvel1[x] × Cam3[t, x] - clm3[x] / (lintest - 2 diflag) bpm3 Cam3[t, x] / xam3a +
  qliv[x] / (lintest - 2 diflag) × Clivm3[t, x] / xam3a -
  qliv[x] / (lintest - 2 diflag) × Cam3[t, x] / xam3a,
D[Cam1[t, x], t] ==
 dif1b[x] × D[Cam1[t, x], {x, 2}] + (ddif1b[x] - vel1[x]) D[Cam1[t, x], x] -
  dvel1[x] × Cam1[t, x] - clm1[x] / (lintest - 2 diflag) bpm1 Cam1[t, x] / xam1a +
  (mwm1 / mwm3) clintm3[x] / (lintest - 2 diflag) × Clivm3[t, x] / xam1a,
D[Cam4[t, x], t] ==
 dif1b[x] × D[Cam4[t, x], {x, 2}] + (ddif1b[x] - vel1[x]) D[Cam4[t, x], x] -
  dvel1[x] × Cam4[t, x] - qliv[x] / (lintest - 2 diflag) bpm4 Cam4[t, x] / xam4a +
  qliv[x] / (lintest - 2 diflag) × Clivm4[t, x] / xam4a -
  clm4[x] / (lintest - 2 diflag) bpm4 Cam4[t, x] / xam4a +
  (mwm4 / mwm4g) clgus[x] × C1m4g[t, x] / xam4a,
D[Cb[t, x], t] ==
 dif1b[x] × D[Cb[t, x], {x, 2}] + (ddif1b[x] - vel1[x]) D[Cb[t, x], x] -
  dvel1[x] × Cb[t, x] + cldb[x] / (lintest - 2 diflag) × Ca[t, x] / xab -
  cldb[x] / (lintest - 2 diflag) × Cb[t, x] / xab, D[Cc[t, x], t] ==
 dif1b[x] × D[Cc[t, x], {x, 2}] + (ddif1b[x] - vel1[x]) D[Cc[t, x], x] -
  dvel1[x] × Cc[t, x] + cldc[x] / (lintest - 2 diflag) × Ca[t, x] / xac -
  cldc[x] / (lintest - 2 diflag) × Cc[t, x] / xac,
D[Cliv[t, x], t] ==
 dif1b[x] × D[Cliv[t, x], {x, 2}] + (ddif1b[x] - vel1[x]) D[Cliv[t, x], x] -
  dvel1[x] × Cliv[t, x] + qliv[x] / (lintest - 2 diflag) bp Ca[t, x] / xaliv -
  qliv[x] / (lintest - 2 diflag) × Cliv[t, x] / xaliv + fg (cli[x] / 2) C3[t, x] / xaliv -
  clinth[x] / (lintest - 2 diflag) × Cliv[t, x] / xaliv -
  clgip[x] / (lintest - 2 diflag) × Cliv[t, x] / xaliv -
```

```
      clgim[x] / (lintest - 2 diflag) × Cliv[t, x] / xaliv,
    D[Clivm3[t, x], t] ==
     dif1b[x] × D[Clivm3[t, x], {x, 2}] + (ddif1b[x] - vel1[x]) D[Clivm3[t, x], x] -
      dvel1[x] × Clivm3[t, x] + qliv[x] / (lintest - 2 diflag) bpm3 Cam3[t, x] / xalivm3 -
      qliv[x] / (lintest - 2 diflag) × Clivm3[t, x] / xalivm3 +
      (mwm3 / mwnal) (0.35) clinth[x] / (lintest - 2 diflag) × Cliv[t, x] / xalivm3 -
      clintm3[x] / (lintest - 2 diflag) × Clivm3[t, x] / xalivm3, D[Clivm4[t, x], t] ==
     dif1b[x] × D[Clivm4[t, x], {x, 2}] + (ddif1b[x] - vel1[x]) D[Clivm4[t, x], x] -
      dvel1[x] × Clivm4[t, x] + qliv[x] / (lintest - 2 diflag) bpm4 Cam4[t, x] / xalivm4 -
      qliv[x] / (lintest - 2 diflag) × Clivm4[t, x] / xalivm4 -
      clintm4g[x] / (lintest - 2 diflag) × Clivm4[t, x] / xalivm4 +
      (mwm4 / mwnal) (0.10) clinth[x] / (lintest - 2 diflag) × Cliv[t, x] / xalivm4,
    D[Cgb[t, x], t] ==
     dif1b[x] × D[Cgb[t, x], {x, 2}] + (ddif1b[x] - vel1[x]) D[Cgb[t, x], x] -
      dvel1[x] × Cgb[t, x] + clgip[x] / (lintest - 2 diflag) × Cliv[t, x] / xagb -
      clgo1 clgo[x] / bdwidth × Cgb[t, x] / xagb -
      clgo2[t] × clgo[x] / bdwidth × Cgb[t, x] / xagb,
    D[Cgbm[t, x], t] ==
     dif1b[x] × D[Cgbm[t, x], {x, 2}] + (ddif1b[x] - vel1[x]) D[Cgbm[t, x], x] -
      dvel1[x] × Cgbm[t, x] + clgim[x] / (lintest - 2 diflag) × Cliv[t, x] / xagb -
      clgo1 clgo[x] / bdwidth × Cgbm[t, x] / xagb -
      clgo2[t] × clgo[x] / bdwidth × Cgbm[t, x] / xagb,
    D[Cgbm4g[t, x], t] == dif1b[x] × D[Cgbm4g[t, x], {x, 2}] +
      (ddif1b[x] - vel1[x]) D[Cgbm4g[t, x], x] - dvel1[x] × Cgbm4g[t, x] +
      (mwm4g / mwm4) clintm4g[x] / (lintest - 2 diflag) × Clivm4[t, x] / xagbm4g -
      clgo1 clgo[x] / bdwidth × Cgbm4g[t, x] / xagbm4g -
      clgo2[t] × clgo[x] / bdwidth × Cgbm4g[t, x] / xagbm4g,
    {Cs1[t, 0] == pulsess1[t], Cs1[0, x] == 0, Cs1[t, 8] == 0, C1[t, 0] == pulseds1[t],
     C1[0, x] == 0, C1[t, 8] == 0, C1m5[t, 0] == 0, C1m5[0, x] == 0, C1m5[t, 8] == 0,
     C1m4g[t, 0] == 0, C1m4g[0, x] == 0, C1m4g[t, 8] == 0, C2[0, x] == 0,
     C2[t, 0] == 0, C2[t, 8] == 0, C3[0, x] == 0, C3[t, 0] == 0, C3[t, 8] == 0,
     C4[t, 0] == 0, C4[0, x] == 0, C4[t, 8] == 0, Ca[0, x] == 0, Ca[t, 0] == 0,
     Ca[t, 8] == 0, Cam5[0, x] == 0, Cam5[t, 0] == 0, Cam5[t, 8] == 0, Cam3[0, x] == 0,
     Cam3[t, 0] == 0, Cam3[t, 8] == 0, Cam1[0, x] == 0, Cam1[t, 0] == 0, Cam1[t, 8] == 0,
     Cam4[0, x] == 0, Cam4[t, 0] == 0, Cam4[t, 8] == 0, Cb[0, x] == 0, Cb[t, 0] == 0,
     Cb[t, 8] == 0, Cc[0, x] == 0, Cc[t, 0] == 0, Cc[t, 8] == 0, Cliv[0, x] == 0,
     Cliv[t, 0] == 0, Cliv[t, 8] == 0, Clivm3[0, x] == 0, Clivm3[t, 0] == 0,
     Clivm3[t, 8] == 0, Clivm4[0, x] == 0, Clivm4[t, 0] == 0, Clivm4[t, 8] == 0,
     Cgb[0, x] == 0, Cgb[t, 0] == 0, Cgb[t, 8] == 0, Cgbm[0, x] == 0, Cgbm[t, 0] == 0,
     Cgbm[t, 8] == 0, Cgbm4g[0, x] == 0, Cgbm4g[t, 0] == 0, Cgbm4g[t, 8] == 0}},
   {Cs1, C1, C1m5, C1m4g, C2, C3, C4, Ca, Cam5, Cam3, Cam1, Cam4, Cb, Cc,
    Cliv, Clivm3, Clivm4, Cgb, Cgbm, Cgbm4g}, {t, 0, 25}, {x, 0, 8},
  Method → {"TimeIntegration" → "IDA",
     "PDEDiscretization" → {"MethodOfLines",
```

```
"DifferentiateBoundaryConditions" → {True, "ScaleFactor" → 40},
"SpatialDiscretization" → {"TensorProductGrid",
  "Coordinates" → {coordx}, "DifferenceOrder" → 3}}}, AccuracyGoal → 4]
```

Out[2221]=

```
{{Cs1 → InterpolatingFunction[ Domain: {{0., 25. }, {0., 8. }} Output: scalar  Data not saved. Save now ],

C1 → InterpolatingFunction[ Domain: {{0., 25. }, {0., 8. }} Output: scalar  Data not saved. Save now ],

C1m5 → InterpolatingFunction[ Domain: {{0., 25. }, {0., 8. }} Output: scalar  Data not saved. Save now ],

C1m4g → InterpolatingFunction[ Domain: {{0., 25. }, {0., 8. }} Output: scalar  Data not saved. Save now ],

C2 → InterpolatingFunction[ Domain: {{0., 25. }, {0., 8. }} Output: scalar  Data not saved. Save now ],

C3 → InterpolatingFunction[ Domain: {{0., 25. }, {0., 8. }} Output: scalar  Data not saved. Save now ],

C4 → InterpolatingFunction[ Domain: {{0., 25. }, {0., 8. }} Output: scalar  Data not saved. Save now ],

Ca → InterpolatingFunction[ Domain: {{0., 25. }, {0., 8. }} Output: scalar  Data not saved. Save now ],

Cam5 → InterpolatingFunction[ Domain: {{0., 25. }, {0., 8. }} Output: scalar  Data not saved. Save now ],
```

Cam3 → InterpolatingFunction[Domain: {{0., 25. }, {0., 8. }} Output: scalar Data not saved. Save now],

Cam1 → InterpolatingFunction[Domain: {{0., 25. }, {0., 8. }} Output: scalar Data not saved. Save now],

Cam4 → InterpolatingFunction[Domain: {{0., 25. }, {0., 8. }} Output: scalar Data not saved. Save now],

Cb → InterpolatingFunction[Domain: {{0., 25. }, {0., 8. }} Output: scalar Data not saved. Save now],

Cc → InterpolatingFunction[Domain: {{0., 25. }, {0., 8. }} Output: scalar Data not saved. Save now],

Cliv → InterpolatingFunction[Domain: {{0., 25. }, {0., 8. }} Output: scalar Data not saved. Save now],

Clivm3 → InterpolatingFunction[Domain: {{0., 25. }, {0., 8. }} Output: scalar Data not saved. Save now],

Clivm4 → InterpolatingFunction[Domain: {{0., 25. }, {0., 8. }} Output: scalar Data not saved. Save now],

Cgb → InterpolatingFunction[Domain: {{0., 25. }, {0., 8. }} Output: scalar Data not saved. Save now],

```
Cgbm → InterpolatingFunction[ Domain: {{0., 25. }, {0., 8. }}
                              Output: scalar
                              Data not saved. Save now ],

Cgbm4g → InterpolatingFunction[ Domain: {{0., 25. }, {0., 8. }}
                                Output: scalar
                                Data not saved. Save now ]}}
```

In[2222]:=

```
plotC1 = Plot3D[C1[t, x] /. soln, {t, 0, 25}, {x, 0, 8},
  PlotRange → {{0, 24}, {0, 7}, {-200, 60 000}}, MaxRecursion → 7]
```

Out[2222]=

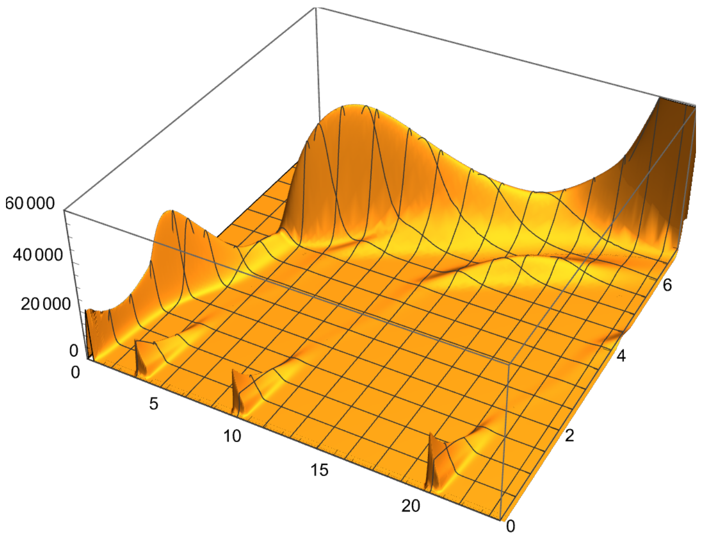

In[2223]:=

```
plotC1m5 = Plot3D[C1m5[t, x] /. soln, {t, 0, 25},
  {x, 0, 8}, PlotRange → {{0, 25}, {0, 8}, {-10, 500 000}},
  AxesLabel → {"t, hr", "x, m", "C, μg/L"}, MaxRecursion → 7]
```

Out[2223]=

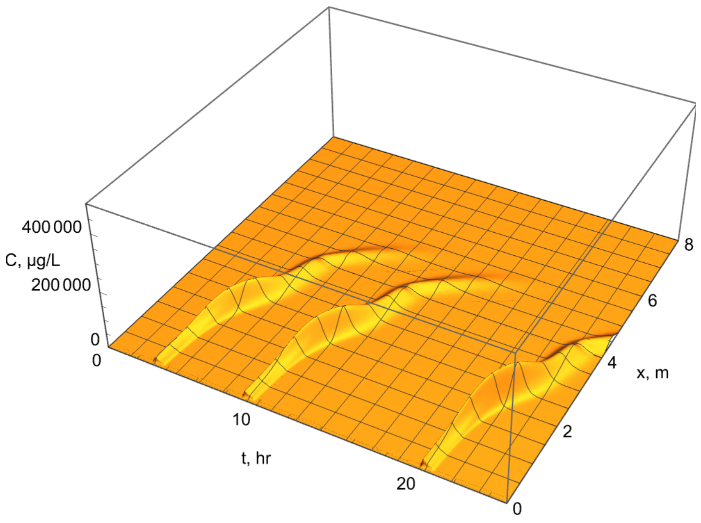

In[2224]:=

```
plotC1m4g = Plot3D[C1m4g[t, x] /. soln, {t, 0, 25},
  {x, 0, 8}, PlotRange → {{0, 25}, {0, 8}, {-10, 500 000}},
  AxesLabel → {"t, hr", "x, m", "C, μg/L"}, MaxRecursion → 7]
```

Out[2224]=

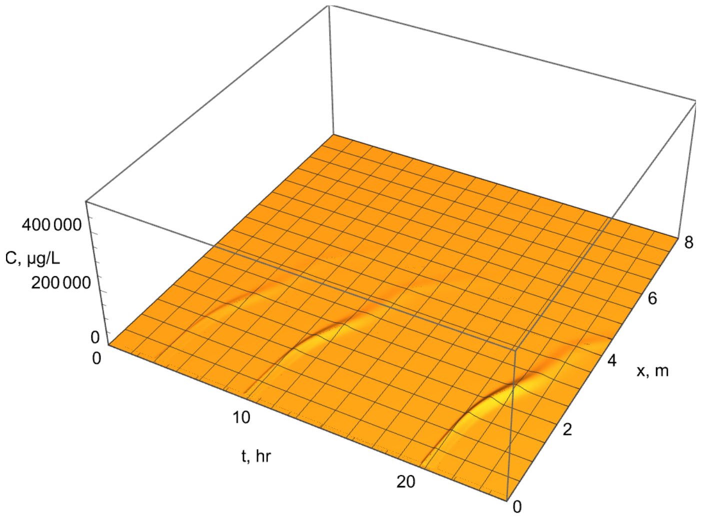

Check Mass Balance

In[2225]:=

```
meth = NIntegrate[(clinth[x] / (lintest - 2 diflag) × Cliv[t, x]) /. soln[[1]],
  {t, 0, 25}, {x, 0, 8}, AccuracyGoal → 10, Method → "AdaptiveQuasiMonteCarlo"]
```

Out[2225]=

```
123.475
```

In[2226]:=
```
metnh = NIntegrate[(clnh[x] / (lintest - 2 diflag) bp Ca[t, x]) /. soln[[1]],
  {t, 0, 25}, {x, 0, 8}, AccuracyGoal → 10, Method → "AdaptiveQuasiMonteCarlo"]
```
Out[2226]=
```
5.07043
```
In[2227]:=
```
residCs1 = NIntegrate[(Cs1[24, x] × xa[x]) /. soln[[1]],
  {x, 0, 8}, AccuracyGoal → 8, Method → "AdaptiveQuasiMonteCarlo"]
```
Out[2227]=
```
4.48616
```
In[2228]:=
```
residC1 = NIntegrate[(C1[24, x] × xa[x]) /. soln[[1]],
  {x, 0, 8}, AccuracyGoal → 7, Method → "AdaptiveQuasiMonteCarlo"]
```
Out[2228]=
```
9.82622
```
In[2229]:=
```
residC1m = NIntegrate[(C1m5[24, x] × xa[x]) /. soln[[1]],
  {x, 0, 8}, AccuracyGoal → 7, Method → "AdaptiveQuasiMonteCarlo"]
```
Out[2229]=
```
9.07651
```
In[2230]:=
```
residC2 = NIntegrate[(C2[24, x] × xamem[x]) /. soln[[1]],
  {x, 0, 8}, AccuracyGoal → 7, Method → "AdaptiveQuasiMonteCarlo"]
```
Out[2230]=
```
0.00342779
```
In[2231]:=
```
residC3 = NIntegrate[(C3[24, x] × xacell[x]) /. soln[[1]],
  {x, 0, 8}, AccuracyGoal → 7, Method → "AdaptiveQuasiMonteCarlo"]
```
Out[2231]=
```
0.00325845
```
In[2232]:=
```
residC4 = NIntegrate[(C4[24, x] × xacell[x]) /. soln[[1]],
  {x, 0, 8}, AccuracyGoal → 7, Method → "AdaptiveQuasiMonteCarlo"]
```
Out[2232]=
```
1.72137
```
In[2233]:=
```
residCa = NIntegrate[(Ca[24, x] xaa) /. soln[[1]],
  {x, 0, 8}, AccuracyGoal → 7, Method → "AdaptiveQuasiMonteCarlo"]
```
Out[2233]=
```
0.373279
```

In[2234]:=
```
residCb = NIntegrate[(Cb[24, x] xab) /. soln⟦1⟧,
  {x, 0, 8}, AccuracyGoal → 7, Method → "AdaptiveQuasiMonteCarlo"]
```
Out[2234]=
```
3.70432
```
In[2235]:=
```
residCc = NIntegrate[(Cc[24, x] xac) /. soln⟦1⟧,
  {x, 0, 8}, AccuracyGoal → 7, Method → "AdaptiveQuasiMonteCarlo"]
```
Out[2235]=
```
0.798922
```
In[2236]:=
```
residCliv = NIntegrate[(Cliv[24, x] xaliv) /. soln⟦1⟧,
  {x, 0, 8}, AccuracyGoal → 7, Method → "AdaptiveQuasiMonteCarlo"]
```
Out[2236]=
```
0.0231025
```
In[2237]:=
```
residCgb = NIntegrate[(Cgb[24, x] xagb) /. soln⟦1⟧,
  {x, 0, 8}, AccuracyGoal → 7, Method → "AdaptiveQuasiMonteCarlo"]
```
Out[2237]=
```
2.76636
```
In[2238]:=
```
residCgbm = NIntegrate[(Cgbm[24, x] xagb) /. soln⟦1⟧,
  {x, 0, 8}, AccuracyGoal → 7, Method → "AdaptiveQuasiMonteCarlo"]
```
Out[2238]=
```
2.76642
```
In[2239]:=
```
total = meth + metnh + residCs1 + residC1 + residC1m + residC2 + residC3 +
  residC4 + residCa + residCb + residCc + residCliv + residCgb + residCgbm
```
Out[2239]=
```
164.095
```
In[2240]:=
```
doseinout =  doseoral / total
```
Out[2240]=
```
0.995156
```

In[2241]:=

```
plotoral1 = LogPlot[Ca[t, 4] /. soln, {t, 0, 25},
  PlotRange → {{0, 25}, {0.1, 100}}, PlotStyle → Black, Frame → True,
  FrameStyle → Directive[Black, 14, Thickness[0.003]],
  LabelStyle → (FontFamily → "Arial"), FrameLabel → {"time,h", "μg/L"}]
```

Out[2241]=

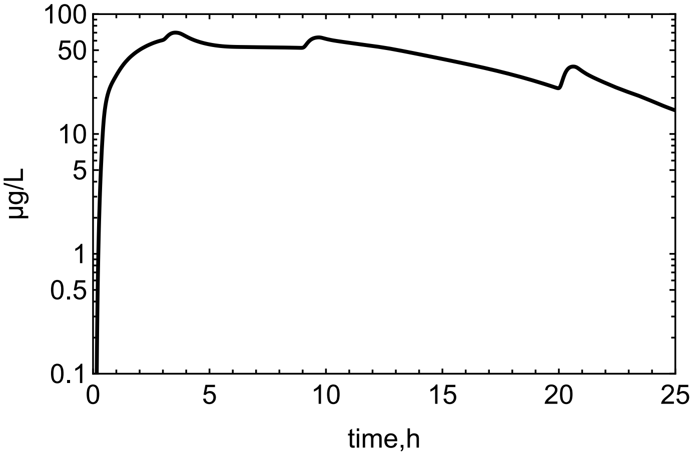

In[2242]:=

```
plotoralm51 = LogPlot[Cam5[t, 4] /. soln, {t, 0, 25},
  PlotRange → {{0, 25}, {0.1, 1000}}, PlotStyle → Black,
  Frame → True, FrameStyle → Directive[Black, 14, Thickness[0.003]],
  LabelStyle → (FontFamily → "Arial"), FrameLabel → {"time,h", "μg/L"}]
```

Out[2242]=

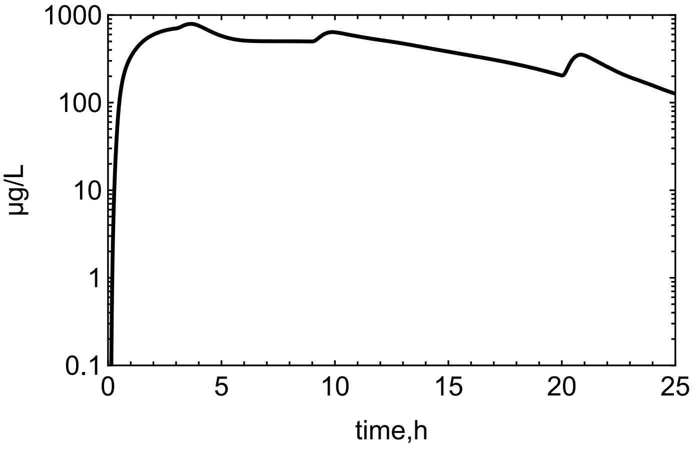

In[2243]:=

```
plotoralm31 = LogPlot[Cam3[t, 4] /. soln, {t, 0, 25},
  PlotRange → {{0, 25}, {0.1, 100}}, PlotStyle → Black, Frame → True,
  FrameStyle → Directive[Black, 14, Thickness[0.003]],
  LabelStyle → (FontFamily → "Arial"), FrameLabel → {"time,h", "μg/L"}]
```

Out[2243]=

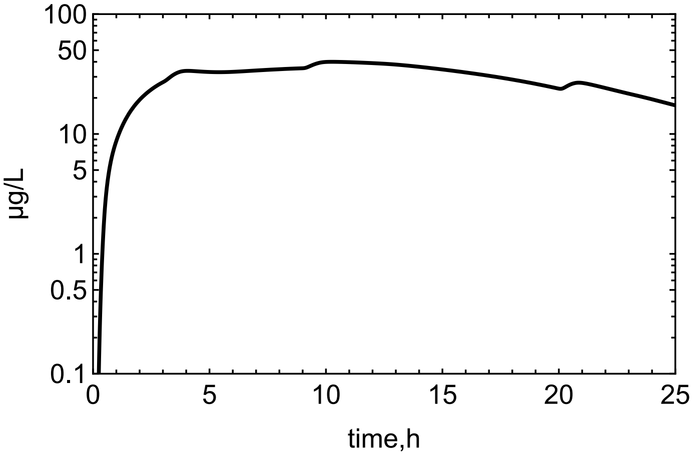

In[2244]:=

```
plotoralm11 = LogPlot[Cam1[t, 4] /. soln, {t, 0, 25},
  PlotRange → {{0, 25}, {0.1, 1000}}, PlotStyle → Black,
  Frame → True, FrameStyle → Directive[Black, 14, Thickness[0.003]],
  LabelStyle → (FontFamily → "Arial"), FrameLabel → {"time,h", "μg/L"}]
```

Out[2244]=

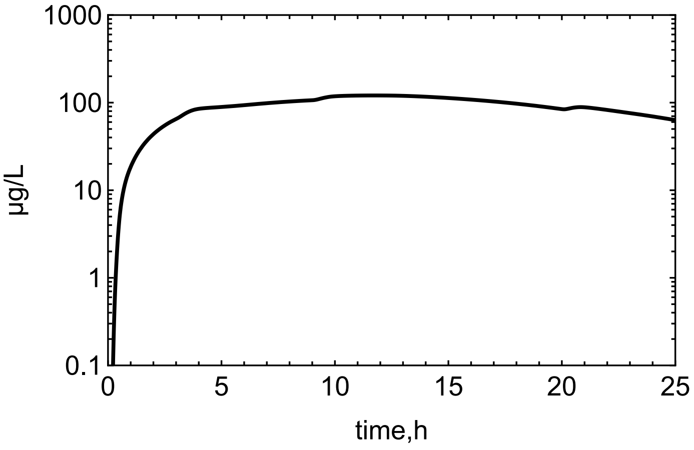

In[2245]:=
```
plotoralm41 = LogPlot[Cam4[t, 4] /. soln, {t, 0, 25},
  PlotRange → {{0, 25}, {0.1, 100}}, PlotStyle → Black, Frame → True,
  FrameStyle → Directive[Black, 14, Thickness[0.003]],
  LabelStyle → (FontFamily → "Arial"), FrameLabel → {"time,h", "μg/L"}]
```
Out[2245]=

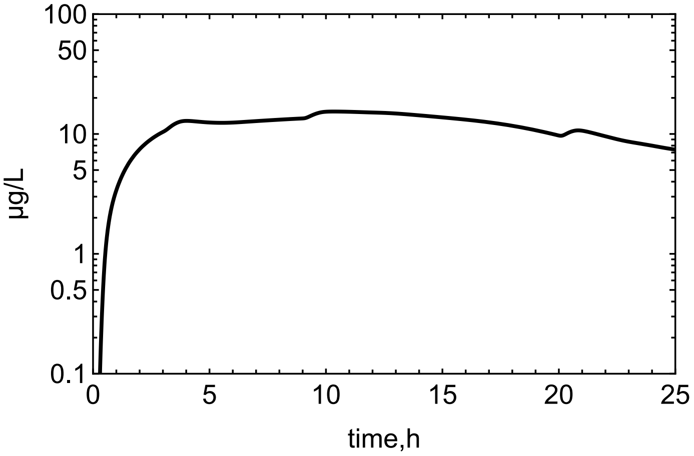

Oral PK inputs

In[2246]:=
```
oralpts = 7;
```
In[2247]:=
```
dataPO = {{1.5, 40.7, 29.9161}, {3., 50.5, 24.2933},
   {5., 56.485, 40.8659}, {7., 58.2167, 40.2894}, {9., 56.4333, 38.8172},
   {12., 56.5167, 31.1061}, {24., 16.5533, 13.4035}};
```
In[2248]:=
```
dataPOsd = Table[{dataPO⟦i, 1⟧, Around[dataPO⟦i, 2⟧, dataPO⟦i, 3⟧]}, {i, 1, oralpts}]
```
Out[2248]=
```
{{1.5, 41. ± 30.}, {3., 51. ± 24.}, {5., 56. ± 41.},
 {7., 58. ± 40.}, {9., 56. ± 39.}, {12., 57. ± 31.}, {24., 17. ± 13.}}
```
In[2249]:=
```
maxpot = 1.1 Max[Table[dataPO⟦i, 1⟧, {i, 1, oralpts}]]
```
Out[2249]=
```
26.4
```
In[2250]:=
```
maxpoc = 1.1 Max[Table[dataPO⟦i, 2⟧, {i, 1, oralpts}]]
```
Out[2250]=
```
64.0384
```

In[2251]:=
```
datam5PO = {{1.5, 496., 253.68},
   {3., 658.667, 202.017}, {5., 680.167, 267.918}, {7., 608.167, 191.79},
   {9., 588.833, 218.627}, {12., 549.5, 235.378}, {24., 138.5, 67.0157}};
```

In[2252]:=
```
datam5POsd =
 Table[{datam5PO⟦i, 1⟧, Around[datam5PO⟦i, 2⟧, datam5PO⟦i, 3⟧]}, {i, 1, oralpts}]
```

Out[2252]=
```
{{1.5, 496. ±254.}, {3., 659. ±202.}, {5., 680. ±268.},
 {7., 608. ±192.}, {9., 589. ±219.}, {12., 550. ±235.}, {24., 139. ±67.}}
```

In[2253]:=
```
maxm5pot = 1.1 Max[Table[datam5PO⟦i, 1⟧, {i, 1, oralpts}]]
```

Out[2253]=
```
26.4
```

In[2254]:=
```
maxm5poc = 1.1 Max[Table[datam5PO⟦i, 2⟧, {i, 1, oralpts}]];
```

In[2255]:=
```
datam3PO = {{1.5, 16.4467, 8.09036},
   {3., 26.3667, 8.24831}, {5., 25.9667, 10.15}, {7., 28.6, 7.50413},
   {9., 30.3, 7.58156}, {12., 32.2167, 11.0648}, {24., 16.145, 5.12985}};
```

In[2256]:=
```
datam3POsd =
 Table[{datam3PO⟦i, 1⟧, Around[datam3PO⟦i, 2⟧, datam3PO⟦i, 3⟧]}, {i, 1, oralpts}]
```

Out[2256]=
```
{{1.5, 16. ±8.}, {3., 26. ±8.}, {5., 26. ±10.},
 {7., 29. ±8.}, {9., 30. ±8.}, {12., 32. ±11.}, {24., 16. ±5.}}
```

In[2257]:=
```
maxm3pot = 1.1 Max[Table[datam3PO⟦i, 1⟧, {i, 1, oralpts}]]
```

Out[2257]=
```
26.4
```

In[2258]:=
```
maxm3poc = 1.1 Max[Table[datam3PO⟦i, 2⟧, {i, 1, oralpts}]];
```

In[2259]:=
```
datam1PO = {{1.5, 4.82, 11.8}, {3., 46.5833, 23.8908},
   {5., 80.0833, 25.2031}, {7., 93.9333, 30.5233},
   {9., 104.167, 27.1857}, {12., 105.617, 44.9775}, {24., 63., 35.2865}};
```

In[2260]:=
```
datam1POsd =
 Table[{datam1PO⟦i, 1⟧, Around[datam1PO⟦i, 2⟧, datam1PO⟦i, 3⟧]}, {i, 1, oralpts}]
```

Out[2260]=
```
{{1.5, 5. ±12.}, {3., 47. ±24.}, {5., 80. ±25.},
 {7., 94. ±31.}, {9., 104. ±27.}, {12., 106. ±45.}, {24., 63. ±35.}}
```

In[2261]:=
```
maxm1pot = 1.1 Max[Table[datam1PO⟦i, 1⟧, {i, 1, oralpts}]]
```

Out[2261]=
```
26.4
```

In[2262]:=
```
maxm1poc = 1.1 Max[Table[datam1PO⟦i, 2⟧, {i, 1, oralpts}]];
```

In[2282]:=
```
datam4PO = {{1.5, 7.82833, 4.72036},
   {3., 12.06, 4.13357}, {5., 12.0767, 3.85312}, {7., 13.2583, 3.32454},
   {9., 13.4033, 3.12052}, {12., 15.1267, 7.49695}, {24., 8.92, 3.60038}};
```

In[2283]:=
```
datam4POsd =
 Table[{datam4PO⟦i, 1⟧, Around[datam4PO⟦i, 2⟧, datam4PO⟦i, 3⟧]}, {i, 1, oralpts}]
```

Out[2283]=
```
{{1.5, 8. ±5.}, {3., 12. ±4.}, {5., 12. ±4.},
 {7., 13.3 ±3.3}, {9., 13.4 ±3.1}, {12., 15. ±7.}, {24., 9. ±4.}}
```

In[2284]:=
```
maxm4pot = 1.1 Max[Table[datam4PO⟦i, 1⟧, {i, 1, oralpts}]]
```

Out[2284]=
```
26.4
```

In[2285]:=
```
maxm4poc = 1.1 Max[Table[datam4PO⟦i, 2⟧, {i, 1, oralpts}]];
```

In[2286]:=
```
plotoralexp = ListLogPlot[dataPOsd,
   PlotRange → {{0, 25}, {0.1, 1.5 maxpoc}}, IntervalMarkers → "Bands"];
```

In[2287]:=

```
Show[ plotoral1, plotoralexp]
```

Out[2287]=

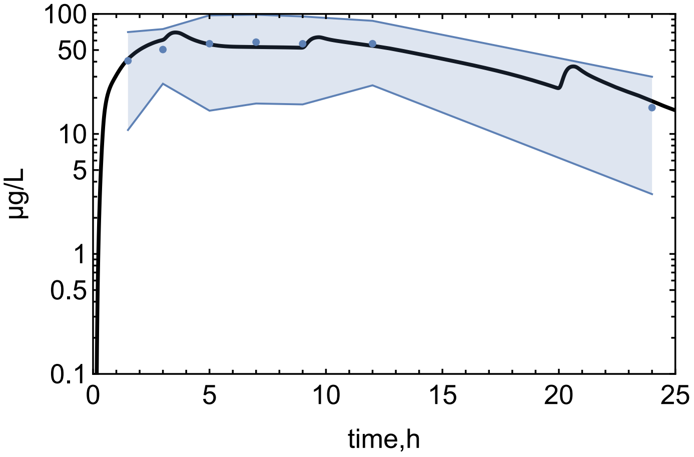

In[2288]:=

```
plotm5oralexp = ListLogPlot[datam5POsd, IntervalMarkers → "Bands"];
```

In[2289]:=

```
Show[plotoralm51, plotm5oralexp]
```

Out[2289]=

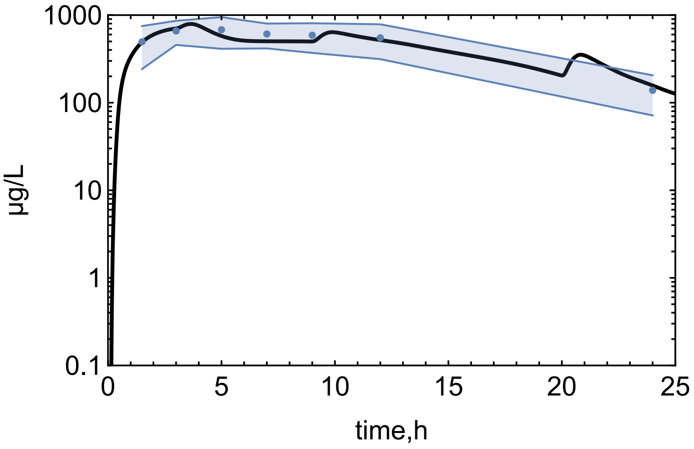

In[2290]:=

```
plotm3oralexp = ListLogPlot[datam3POsd, IntervalMarkers → "Bands"];
```

In[2291]:=

```
Show[plotoralm31, plotm3oralexp]
```

Out[2291]=

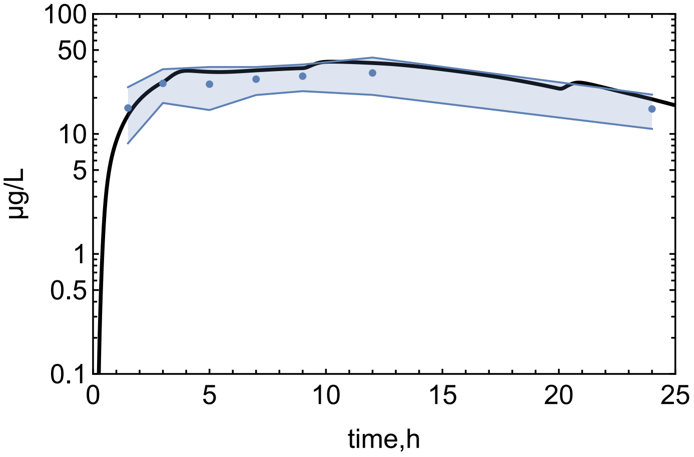

In[2292]:=

```
plotm1oralexp = ListLogPlot[datam1POsd, IntervalMarkers → "Bands"];
```

In[2293]:=

```
Show[plotoralm11, plotm1oralexp]
```

Out[2293]=

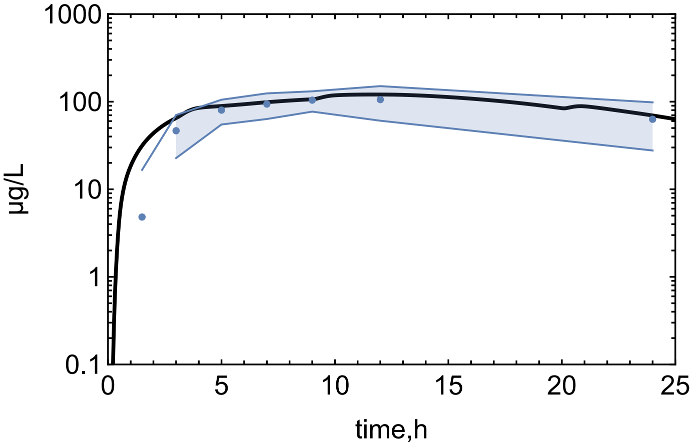

In[2294]:=

```
plotm4oralexp = ListLogPlot[datam4POsd, IntervalMarkers → "Bands"];
```

In[2295]:=

```
Show[plotoralm41, plotm4oralexp]
```

Out[2295]=

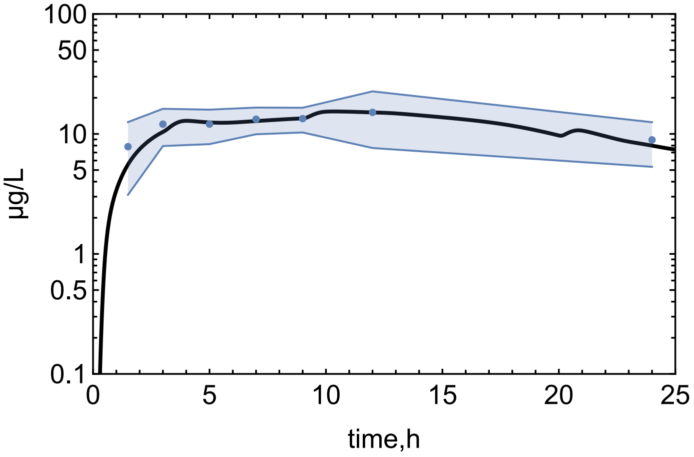

In[2277]:=

```
aucNALb = NIntegrate[(Ca[t, 4]) /. soln⟦1⟧,
  {t, 0, 24}, AccuracyGoal → 7, Method → "AdaptiveQuasiMonteCarlo"]
```

Out[2277]=

```
1045.7
```

In[2278]:=

```
aucM5b = NIntegrate[(Cam5[t, 4]) /. soln⟦1⟧,
  {t, 0, 24}, AccuracyGoal → 7, Method → "AdaptiveQuasiMonteCarlo"]
```

Out[2278]=

```
10 267.
```

In[2279]:=

```
aucM3b = NIntegrate[(Cam3[t, 4]) /. soln⟦1⟧,
  {t, 0, 24}, AccuracyGoal → 7, Method → "AdaptiveQuasiMonteCarlo"]
```

Out[2279]=

```
710.474
```

In[2280]:=

```
aucM1b = NIntegrate[(Cam1[t, 4]) /. soln⟦1⟧,
  {t, 0, 24}, AccuracyGoal → 7, Method → "AdaptiveQuasiMonteCarlo"]
```

Out[2280]=

```
2178.83
```

In[2281]:=

```
aucM4b = NIntegrate[(Cam4[t, 4]) /. soln⟦1⟧,
  {t, 0, 24}, AccuracyGoal → 7, Method → "AdaptiveQuasiMonteCarlo"]
```

Out[2281]=

```
277.994
```
